# CopG_1_, a Novel Transcriptional Regulator Affecting Symbiosis in *Bradyrhizobium* sp. SUTN9-2

**DOI:** 10.3390/biology13060415

**Published:** 2024-06-05

**Authors:** Praneet Wangthaisong, Pongdet Piromyou, Pongpan Songwattana, Tarnee Phimphong, Apisit Songsaeng, Natcha Pruksametanan, Pakpoom Boonchuen, Jenjira Wongdee, Kamonluck Teamtaisong, Nantakorn Boonkerd, Shusei Sato, Panlada Tittabutr, Neung Teaumroong

**Affiliations:** 1 School of Biotechnology, Institute of Agricultural Technology, Suranaree University of Technology, Nakhon Ratchasima 30000, Thailand; 2 Institute of Research and Development, Suranaree University of Technology, Nakhon Ratchasima 30000, Thailand; 3 The Center for Scientific and Technological Equipment, Suranaree University of Technology, Nakhon Ratchasima 30000, Thailand; 4 Graduate School of Life Sciences, Tohoku University, Sendai 980-8577, Japan

**Keywords:** symbiosis, *Vigna radiata*, T4SS, *copG*

## Abstract

**Simple Summary:**

In the process of symbiosis, Δ*copG_1_* in the type IV secretion system (T4SS) demonstrated the ability to invade root cells but was unable to survive and multiply within root cells. Conversely, *traG_1_* and *virD2_1_* were found to be essential in the early stages of nodule formation. Intriguingly, *copG_1_* is required for nod gene expression and acts as a repressor of T4SS genes. Moreover, the absence of *copG_1_* results in certain proteins not being produced, especially T3SS (*nopX* and *nopP*) and C_4_-dicarboxylic acid (*dct*), which affects the symbiosis between *Bradyrhizobium* sp. SUTN9-2 and legumes. These findings support the hypothesis that the *copG_1_* gene may serve as a new regulator of the symbiotic process.

**Abstract:**

The symbiotic interaction between leguminous and *Bradyrhizobium* sp. SUTN9-2 mainly relies on the nodulation process through Nod factors (NFs), while the type IV secretion system (T4SS) acts as an alternative pathway in this symbiosis. Two copies of T4SS (T4SS_1_ and T4SS_2_) are located on the chromosome of SUTN9-2. ΔT4SS_1_ reduces both nodule number and nitrogenase activity in all SUTN9-2 nodulating legumes. The functions of three selected genes (*copG_1_*, *traG_1_*, and *virD2_1_*) within the region of T4SS_1_ were examined. We generated deleted mutants and tested them in *Vigna radiata* cv. SUT4. Δ*traG_1_* and Δ*virD2_1_* exhibited lower invasion efficiency at the early stages of root infection but could be recently restored. In contrast, Δ*copG_1_* completely hindered nodule organogenesis and nitrogenase activity in all tested legumes. Δ*copG_1_* showed low expression of the nodulation gene and *ttsI* but exhibited high expression levels of the T4SS genes, *traG_1_* and *trbE_1_*. The secreted proteins from ΔT4SS_1_ were down-regulated compared to the wild-type. Although *ΔcopG_1_* secreted several proteins after flavonoid induction, T3SS (*nopP* and *nopX*) and the C_4_-dicarboxylate transporter (*dct*) were not detected. These results confirm the crucial role of the *copG_1_* gene as a novel key regulator in the symbiotic relationship between SUTN9-2 and legumes.

## 1. Introduction

Rhizobia-legume symbiosis is a key process of mutually beneficial relationships where nitrogen-fixing Rhizobia bacteria form root nodules and convert atmospheric nitrogen into ammonia that can be used by the plant, while the plant provides the bacteria with carbohydrates [1]. This symbiosis is ecologically significant for providing a major input of nitrogen into ecosystems. It also benefits agriculture by reducing reliance on synthetic nitrogen fertilizers, which can have negative environmental impacts [2]. However, specific symbiotic relationships between rhizobia and plant species require a complex exchange of signaling compounds, which is the key factor in successful symbiosis [3]. Two crucial mechanisms are necessary for the nodulation process of many rhizobia: (i) the classical mechanism of perception of the Nod factors (NFs), which are lipochitooligosaccharides (LCOs) produced by nitrogen-fixing rhizobia. These signal molecules are essential components for successful symbiosis [4]. (ii) secretion systems, the mechanism utilized mainly by pathogens to deliver effector proteins into their hosts [5]. For plant-microbe interaction, effector proteins are recognized by resistance (R) proteins, triggering rapid defense responses called effector-triggered immunity (ETI) and causing a hypersensitive response (HR) to prevent pathogen invasion and disease [6,7]. Nevertheless, the secretion systems have become one of the key mechanisms for the successful symbiosis of many rhizobia with legumes.

Secretion systems, including type III (T3SS), type IV (T4SS), and type VI (T6SS), have been identified in various genera of rhizobia. These systems are also reported as the key determinants of symbiotic interactions during infection processes, and the T3SS is the most prevalent secretion system among rhizobia [8,9,10]. Effector proteins are secreted into the host plant to trigger effector-triggered susceptibility (ETS), enabling successful infection and survival in the host [11]. Remarkably, the *Rhizobium* harboring secretion systems and effectors demonstrates the ability to suppress host defenses and facilitate infection, similar to plant-pathogen interactions [12]. The secreted effector proteins from T3SS (T3Es) can have either a positive or negative impact on symbiosis efficiency, depending on the plant species [12]. For example, *Bradyrhizobium vignae* ORS3257 contains multiple effector proteins that are crucial for modulating symbiotic properties in different *Vigna* species. NopT and NopAB play essential roles in nodulation in *V. unguiculata* and *V. mungo*. Whereas, NopP2 displayed incompatibility with *V. radiata* [13]. Beside the T3SS, the T6SS of *Rhizobium etli* Mim1 and *Bradyrhizobium* sp. LmicA16 (A16) exhibit a positive effect on nodulation with their host [14,15]. In the case of T4SS, this secretion machinery is found in various bacteria. It functions as a molecular channel, allowing bacteria to transport diverse molecules across their cell envelope [16]. Generally, T4SS is utilized by *Agrobacterium* sp. to transfer T-DNA into plant hosts, causing crown gall disease [17]. Various rhizobia, including *Bradyrhizobium*, *Rhizobium*, *Sinorhizobium*, and *Mesorhizobium*, possess T4SS homologs to those found in *Agrobacterium* sp. These systems belong to the *tra/trb* operon and can be located either in the chromosome or plasmid, depending on the bacterial strain [18,19,20,21]. Interestingly, most bradyrhizobia harbor the *tra*/*trb* operon on the chromosome, but a few studies on the role of T4SS of *Bradyrhizobium* in the symbiotic process have been reported. 

This study focuses on *Bradyrhizobium* sp. SUTN9-2, a broad host range strain known for its diverse ability to nodulate with various legumes. Moreover, it acts as a rice endophyte, playing a crucial role in promoting rice growth [22,23]. Previously, we found two clusters of T4SS (T4SS_1_ and T4SS_2_) located on the chromosome of SUTN9-2 with different gene arrangements (Figure S1). A specialized gene arrangement consisting of *copG* (a putative transcriptional factor), *traG* (T4SS structural), and *virD2* (relaxase) was observed in the T4SS gene cluster of *Bradyrhizobium* species. The T4SS evolutionary analysis of the *Rhizobiales* order, encompassing *Bradyrhizobium*, *Rhizobium*, *Sinorhizobium*, and *Mesorhizobium* through the *traG* gene phylogenetic tree, demonstrates a co-evolutionary trend between *Bradyrhizobium* and *Mesorhizobium*. Upon phylogenetic examination of the *copG*, *traG*, and *virD2* combination genes in *Bradyrhizobium*, two copies of these clusters were divided into two clades within the bradyrhizobia group [24]. Moreover, copy 1 of the *copG*, *traG*, and *virD2* genes exhibits a close evolutionary association with *B. yuanmingense* BRP09, the main rhizobia associated with cowpea and mung bean in the subtropical region of China [25], as well as with *B. diazoefficiens* USDA110, a soybean inoculant [26]. Interestingly, the T4SS_1_ mutant (cluster deletion including *copG_1_*, *traG_1_*, and *virD2_1_* fragment) retarded nodulation ability in *V. radiata* cv. SUT4 and *Crotalaria juncea,* and both the number of nodules and nitrogenase activity were decreased compared with the wild-type. The results indicated that T4SS_1_ has a positive effect on symbiotic interactions with the tested plants [24]. To elucidate the role of T4SS_1_ in symbiosis, this study investigated the functions of individual *copG_1_*, *traG_1_*, and *virD2_1_* genes during their interaction with *V. radiata* cv. SUT4. Notably, this study reveals a crucial function of *copG_1_* in regulating symbiosis not only in *V. radiata* but potentially across the Genistoids, Dalbergioids, and Millettioids lineages. This knowledge could be applied to develop future rhizobial inoculants that enhance nitrogen fixation capabilities in a wide range of legumes.

## 2. Materials and Methods

### 2.1. Bacterial Strains, Plasmids, and Growth Conditions

The bacterial strains and plasmids used in this study are listed in Table S1. *Bradyrhizobium* sp. SUTN9-2 was grown in an arabinose-gluconate (AG) medium [27]. The derivative mutants Δ*copG_1_*, Δ*traG_1_*, and Δ*virD2_1_* were supplemented with streptomycin (sm) at 200 µg/mL. *Escherichia coli* strains were grown at 37 °C in Luria-Bertani (LB) medium. Antibiotics were added to the medium as required at the following concentrations: 50 µg/mL kanamycin (km), 30 µg/mL nalidixic acid (nal), and 200 µg/mL streptomycin (sm).

### 2.2. Plasmid Construction and Gene Deletion

The deletion mutants of *copG_1_*, *copG_2_*, *traG_1_*, and *virD2_1_* genes in *Bradyrhizobium* sp. SUTN9-2 (GeneBank accession number LAXE00000001) were obtained as follows: The upstream and downstream regions of *copG_1_* (up: 575 bp, dw: 841 bp), *copG_2_* (up: 874 bp, dw:1043 bp), *traG_1_* (up: 1060 bp, dw:921 bp), and *virD2_1_* (up: 944 bp, dw: 738 bp) genes were obtained by PCR using the primers listed in Table 1. The target deletion genes in SUTN9-2 were obtained by double crossover. PCR fragments corresponding to the upstream and downstream flanking regions of the gene of interest were merged by overlap extension and introduced into a pNTPS129 plasmid harboring the *sacB* gene [28]. Then, an Ω cassette fragment (spectinomycin/streptomycin resistance genes) from pHP45 (omega) [29] was introduced between the upstream and downstream flanking regions, which were already cloned into pNTPS129. The restriction sites for antibiotic insertion were *HindIII* for *copG_1_* and *BamHI* for *copG_2_*, *traG_1_*, and *virD2_1_*. The recombinant plasmids were transferred into SUTN9-2 by triparental mating using pRK2013 as a helper plasmid [30], as described previously [24]. A single recombinant clone was obtained from antibiotic selection and PCR verification. Double recombinant clones were selected by culture on AG medium supplemented with 10% sucrose and 200 µg/mL sm. Candidate clones were verified for the loss of the *sacB* gene from pNTPS129, and the replacement of the Ω cassette was verified by PCR. All mutant strains were further investigated for nodulation efficiency in *V. radiata* cv. SUT4.

### 2.3. Nodulation Test and Acetylene Reduction Assay (ARA)

*V. radiata* cv. SUT4 seeds were surface sterilized and germinated as previously described [32] and placed on 0.85% water agar at 28 °C overnight. One-day-old germinated seedlings were transferred into Leonard’s jars containing sterilized vermiculite and liquid buffered nodulation media (BNM) [33]. Seven days after gemination, seedlings were inoculated with a bacterial suspension of *Bradyrhizobium* sp. SUTN9-2 or derivative mutants (1 mL per seedling; adjusted to OD_600_ = 0.8). Five plants per treatment were selected for nodule counting. Symbiotic phenotypes and nitrogen activity were measured at 7, 14, and 21 dpi.

Acetylene reduction assays (ARAs) were used to evaluate nitrogenase activity. The root samples were transferred into test tubes, which were closed with a plastic stopper. The samples were then incubated with 10% (*v*/*v*) pure acetylene instead of air, which was withdrawn for 1 h at room temperature. A 1 mL sample was examined using gas chromatography (GC) with a PE-alumina-packed column to measure the conversion of acetylene (C_2_H_2_) to ethylene (C_2_H_4_). Detection was performed at an injection temperature of 150 °C and oven temperatures of 200 °C and 50 °C for flame ionization detection (FID) [34]. The experiment was conducted with five biological replicates per treatment. Nitrogenase activity is presented in nmol ethylene/h/plant dry weight [35].

The symbiotic profiles of Δ*copG_1_* were compared with those of SUTN9-2 using a growth pouch test and several leguminous plants, including Genistoids (*Crotalaria juncea*), Dalbergioids (*Aeschynomene americana* cv. Thai, *Arachis hypogaea* cv. Thainan 9 and *A. hypogaea* cv. Khonkaen 5), and Millettioids (*Indigofera tinctoria*, *Macroptilium atropurpureum*, *V. radiata* cv. SUT1, *V. radiata* cv. CN72, *V. radiata* cv. KUML4, *V. radiata* cv. CN36, *V. radiata* cv. KPS1, *V. mungo* cv. U thong 2 and, *V. subterranean*). Seeds were sterilized and germinated as previously described [32,36]. Pouches were prepared [35] and supplemented with BNM medium. Seedlings were grown at two plants per pouch (Five pouch replicates per treatment) and inoculated with 1 mL per plant of a suspension containing OD_600_ = 0.8. Plants were grown under the conditions mentioned above.

### 2.4. Bacterial Induction, RNA Isolation, and qRT–PCR Analysis of Gene Expression

For bacterial induction, the mid-log phase of bacterial cultures, including *Bradyrhizobium* sp. SUTN9-2 and *copG_1_* mutant strains (OD_600_ = 0.4), was induced by 20 μM genistein at 28 °C for 24 h. Then, bacterial pellets were collected by centrifugation (4000× *g*, at 4 °C) for total RNA isolation. Total RNA was isolated from bacterial pellets using an RNeasy^®^ Protect Cell Mini Kit (Qiagen, Chatsworth, CA, USA) according to the manufacturer’s instructions. Total RNA was treated at 37 °C for 30 min with RNase-free DNase I (New England Biolabs, Ipswich, MA, USA). 

cDNA was synthesized using iScript™ Reverse Transcription Supermix for RT-qPCR (Bio-Rad Laboratories, Inc., Hercules, CA, USA) according to the manufacturer’s protocol. A cDNA concentration of 50 ng/µL was subjected to real-time PCR using specific primers (Table 1) for nodulation genes (*nodA*, *nodB*, *nodC*, *nodD1*, and *nodD2*), transcriptional regulator of T3SS (*ttsI*), T4SS structural genes (*traG_1_* and *trbE_1_*), and other genes. qRT-PCR reactions were performed with Luna^®^ Universal qPCR Master Mix (NEB, Ipswich, MA, USA) according to the manufacturer’s protocol, and thermal cycling was conducted in a CFX Opus 96 Real-Time PCR System (Bio-Rad Laboratories, Inc.). The reactions were performed in triplicate for each of the three biological replicates. Relative gene expression was analyzed by the comparative Ct method 2(−ΔΔCT), and 16s rRNA (accession number: JN578804) was used as an internal control [18]. Three biological replicates were analyzed.

### 2.5. Protein Preparation, Sodium Dodecyl Sulfate Polyacrylamide Gel Electrophoresis (SDS-PAGE) Analysis, and Protein Identification

Wild-type (WT) and Δ*copG_1_ Bradyrhizobium* sp. SUTN9-2 were grown in AG medium with shaking at 200 rpm and 30 °C until they reached an OD_600_ of 1. One percent (*v*/*v*) of each starter was inoculated into 100 mL of AG medium with and without 20 µM genistein induction. The cultures were then incubated at 30 °C until they reached an OD_600_ of approximately 1.0. The bacterial supernatants were harvested by centrifugation at 4000× *g* and 4 °C for 1 h, followed by 8000× *g* for 30 min. One milliliter of 1 M dithiothreitol (DTT) and 7.5 mL of phenol solution (equilibrated with 10 mM Tris HCl at pH 8.0 with ESTA) were added into 25 mL of fresh supernatant. The solution was vigorously mixed with a vortex before centrifugation at 8000× *g* and 4 °C for 30 min. The water phase was discarded, and the phenol phase was added into another 25 mL of supernatant, followed by vigorous mixing and centrifugation at 8000× *g* and 4 °C for 30 min. Next, 20 mL of methanol containing 300 µL of 8 M ammonium acetate and 400 µL of 1 M dithiothreitol were added to remove the phenol phase. The secreted protein was precipitated overnight at 20 °C. The solution was then centrifuged at 8000× *g* and 4 °C for 1 h, and the supernatant was discarded. After precipitation, the protein was washed with chilled 70% (*v*/*v*) ethanol and air-dried in a laminar flow before being dissolved in phosphate-buffered saline (PBS). Protein concentrations were determined using a plate reader and the manufacturer’s protocol (PanReac, Barcelona, Spain) according to the Bradford method [37]. A standard calibration curve was constructed using 0 to 2 µg of bovine serum albumin (BSA). Denaturing SDS-PAGE was performed according to the method of Laemmli [38], in which 10 µg of each lane of protein was analyzed on a 12% SDS-PAGE gel. The protein samples were mixed with loading buffer containing β-mercaptoethanol and heated for 10 min before loading. Protein bands were stained with colloidal Coomassie brilliant blue R-250 to visualize the expression of secreted protein. The protein bands that were observed on the WT lane but not observed on the ∆T4SS_1_ lane were cut for protein identification by mass spectrometry. Breiftly, the protein bands were performed ingel digestion by 12.5 ng/µL trypsin (mass spectrometry grade; Promega, Madison, WI, USA). The extracted peptides were collected and dried in the Nitrogen Evaporator (Organomation, Berlin, MA, USA). The peptides were then reconstituted in 15 µL of 0.1% formic acid (FA) for LC/MS analysis. The LC-MS/MS system consists of a liquid chromatography part (Dionex Ultimate 3000, RSLCnano System, Thermo Fisher Scientific, Waltham, MA, USA) in combination with a captivespray ionization/mass spectrometer (Model Q-ToF Compact, Bruker, Germany) at the Proteomics Services, Faculty of Medical Technology, Mahidol University (Salaya Campus, Mahidol University, Nakhon Pathom, Thailand). Mass spectral data from 300 to 1500 m/z were collected in the positive ionization mode. The most abundant peptide ions were analyzed using MS/MS to determine the peptide sequence. The peptide sequence was searched on the UniProt database using the Mascot Daemon version 2.6.0 (Matrix Science, London, UK) search engine. The search parameters in the Mascot daemon MS/MS Ions search included carbamidomethyl at cysteine residues as a fixed modification and oxidation on methionine as a variable modification. The peptide tolerance was set at ±1.6 Da, and the MS/MS fragment tolerance was set at ±0.8 Da. Protein hits were selected with a *p*-value of ≤0.05. The obtained results were examined against the protein-NCBI database to identify and annotate proteins.

### 2.6. Microscopy

Nodule phenotypes and cross sections of representative nodules generated by the wild-type (WT) or mutants were examined under a stereomicroscope LEIGA EZ4 (Leica Microsystems, Wetzlar, Germany). For in-situ live or dead cell staining, the nodules were harvested and embedded in 5% agarose [39]. Three plants per treatment were selected for nodule sections with a thickness of 40–50 µm. They were prepared with a VT1000S vibratome (Leica, Nanterre, France) and incubated with live/dead staining solution (5 µM SYTO9 and 30 µM propidium iodide (PI) in PBS pH 7.0 buffer) for 30 min, followed by staining with 1 calcofluor-white stain for 20 min. Sections were washed to remove the staining solution and mounted in 10% glycerol in PBS buffer. After staining, nodules were observed by confocal microscopy using a Nikon Inverted Eclipse Ti-E Confocal Laser Scanning Microscope. Calcofluor-white was detected with emission at 460–500 nm, while SYTO9 and PI were detected at 510–570 nm and 600–650 nm, respectively [24]. Three nodules were randomly selected for imaging and bacteroid observation.

### 2.7. Bioinformatics

*Bradyrhizobium* sp. SUTN9-2 genome sequences were obtained from the NCBI database (https://www.ncbi.nlm.nih.gov, accessed on 9 November 2021) and Genoscope (https://mage.genoscope.cns.fr, accessed on 15 February 2022) [40]. Multiple sequence alignments were determined using CLUSTALW (2.1) (https://www.genome.jp/tools-bin/clustalw, accessed on 20 April 2023). Domain architecture analysis was performed using the Simple Modular Architecture Research Tool (SMART) (https://smart.embl.de, accessed on 31 March 2023) [41] and InterPro (https://www.ebi.ac.uk/interpro, accessed on 20 April 2023) [42]. The annotation features and whole genome sequences were analyzed using SnapGene software version 7.2.0 (www.snapgene.com, accessed on 20 April 2023). 

### 2.8. Statistical Analysis

All data were obtained from experiments performed in triplicate. For statistical analyses, one-way analysis of variance (ANOVA) followed by Tukey’s honestly significant difference (HSD) test (Tukey’s tests at *p* ≤ 0.05) and Student’s *t*-tests (*p* ≤ 0.05) were performed using SPSS software (SPSS version 22.0 windows: SPSS Inc., Chicago, IL, USA) and GraphPad Prism statistical software (Version 10.0.3).

## 3. Results

### 3.1. Symbiotic Properties of ΔcopG_1_, ΔtraG_1_, and ΔvirD2_1_ in Vigna radiata cv. SUT4 

**Figure 1:**
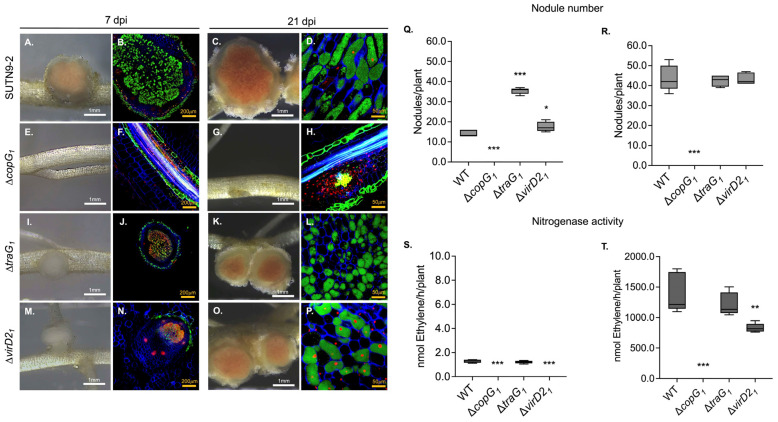
Symbiotic phenotypes of *Bradyrhizobium* sp. SUTN9-2 mutants during symbiosis with *Vigna radiata* cv. SUT4. Nodule phenotype at 7 and 21 dpi were generated by wild-type (**A**,**C**), Δ*copG_1_* (**E**,**G**), Δ*traG_1_* (**I**,**K**), and Δ*virD2_1_* (**M**,**O**). Cytological analysis of the nodules (at 7 dpi and 21 dpi) induced by SUTN9-2 with wild-type (**B**,**D**), Δ*copG_1_* (**F**,**H**), Δ*traG_1_* (**J**,**L**), and Δ*virD2_1_* (**N**,**P**) observed by confocal microscopy after staining with propidium iodide, PI (red; infected plant nuclei and dead bacteria), SYTO9 (green: live bacteria), and calcofluor-white (blue: plant cell wall). Number of nodules at 7 dpi (**Q**) and 21 dpi (**R**). Nitrogen fixation activity was determined by the acetylene reduction assay (ARA) of plants infected with the indicated bacterial mutants at 7 dpi (**S**) and 21 dpi (**T**). Values represent the mean ± SD (*n* = 5). Scale bars: white bars indicate 1 mm, yellow bars indicate 100 µm (20X), and 50 µm (40X). *p* values based on Tukey’s test (* *p* < 0.05, ** *p* < 0.01, *** *p* < 0.001).

Differences between the wild-type and mutants of *Bradyrhizobium* sp. SUTN9-2 were observed in terms of nodulation and nitrogenase activity in *V. radiata* cv. SUT4 (Figure 1). Δ*traG_1_* and Δ*virD2_1_* induced a higher number of nodules on the plant tested than the wild-type at 7 days post-inoculation (dpi) (Figure 1Q), although the nodules produced displayed a white color (Figure 1I,M) instead of pink, indicating problems in nodule development. Moreover, there were higher numbers of dead cells in nodules inoculated with Δ*traG_1_* and Δ*virD2_1_* in the symbiosome area (Figure 1J,N). At 21 dpi, there was no difference in the number of nodules obtained using Δ*traG_1_*, Δ*virD2_1_*, or the wild-type (Figure 1R). 

In addition, there were different results of nitrogenase activities in each mutant at 21 dpi; Δ*traG_1_* was identical to that of the wild-type, whereas low nitrogenase activity was obtained with Δ*virD2_1_* (Figure 1T). Interestingly, Δ*copG_1_* showed a significant effect on nodulation in that nodule formation was abolished (Figure 1E,G,Q,R). Although nodule organogenesis was not observed in the plant inoculated with Δ*copG_1_*, both live and dead cells were detected in the cortex and vascular tissue instead (Figure 1H). According to the results, Δ*copG_1_* could infect plant cells, but it was no longer capable of surviving in host cells. 

Interestingly, *copG_1_* has the potential to regulate symbiosis not only in *V. radiata* but also across diverse lineages such as Genistoids, Dalbergioids, and Millettioids (Table S2). These findings suggest that *copG_1_* may have a conserved role in governing symbiosis interactions across various plant species by controlling the primary symbiotic interaction system.

### 3.2. The copG Genes Are Involved in Nodulation Efficiency of Bradyrhizobium sp. SUTN9-2

The *copG* gene typically encodes the CopG protein, which is a transcription factor consisting of ribbon helix-turn-helix (RHH) motifs. *Bradyrhizobium* sp. SUTN9-2 has two copies of the *copG* gene located downstream of the *traG* and *virD2* genes. These gene clusters are located in distinct locations on the SUTN9-2 chromosome. Although two copies of the *copG* genes were present on the chromosome, they did not share the same gene sequences. The *copG* gene copies 1 and 2 (*copG_1_* and *copG_2_*) revealed a low degree of similarity, with 52.35% DNA sequence identity (Figure S2) and 51.77% amino acid sequence identity (Figure S3), which differ in both the N- and C-terminals. Domain architecture analysis identified CopG_1_ as an unidentified domain, which shows similarities with *B. yuanmingense* BRP09, CCBAU05623, and, more distantly, in P10 130. While, CopG_2_ was identified as a Pfam:RHH domain that is also found in *B. diazoefficiens* USDA110, *B. diazoefficiens* SEMIA5080, and *B. japonicum* J5 (Figure S4). 

**Figure 2:**
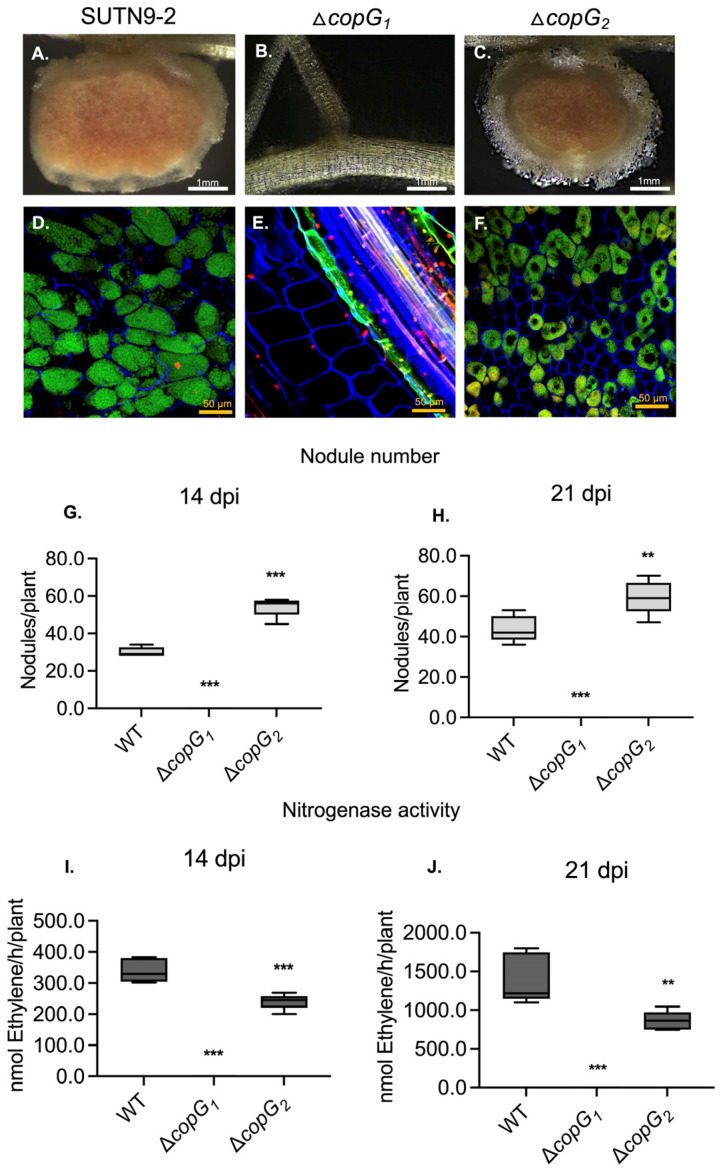
Derivative *copG* mutants of *Bradyrhizobium* sp. SUTN9-2 exhibit different symbiotic interactions with *Vigna radiata* cv. SUT4. Nodule phenotypes induced by wild-type (**A**), Δ*copG_1_* (**B**), and Δ*copG_2_* (**C**). Cytological analysis of live and dead cells of section nodules infected with wild-type (**D**), Δ*copG_1_* (**E**), and Δ*copG_2_* (**F**) at 14 dpi was performed using confocal microscopy, and bacteroids were stained with PI (red; infected plant nuclei and dead bacteria), SYTO9 (green: live bacteria), and calcofluor-white (blue: plant cell wall). Number of nodules at 14 dpi (**G**) and 21 dpi (**H**). Nitrogen fixation activity was determined by the ARA of plants infected with the indicated bacterial mutants at 14 (**I**) and 21 dpi (**J**). Values represent the mean ± SD (*n* = 5). Scale bars: white bars indicate 1 mm, and yellow bars indicate 50 µm (40X). *p* values based on Tukey’s test (** *p* < 0.01, *** *p* < 0.001).

To gain a more complete understanding of the functions of *copG_1_* and *copG_2_*, we constructed Δ*copG_2_*, inoculated it into *V. radiata* cv. SUT4, and compared it with Δ*copG_1_* and wild-type strains (Figure 2). Contrary to Δ*copG_1_* (Figure 2B), Δ*copG_2_* was able to produce pink nodules (Figure 2C) that were smaller than those generated by the wild-type (Figure 2A). At 14 and 21 dpi, Δ*copG_2_* produced the highest number of nodules compared with the other strains (Figure 2G,H). Despite the high number of nodules generated by Δ*copG_2_*, the nitrogenase activity was significantly lower than that of the wild-type (Figure 2I,J). Confocal microscopic examination showed dead cells in nodules generated by Δ*copG_2_* (Figure 2F) in the symbiosome, as seen in red after staining with PI, in contrast with the wild-type (Figure 2D), which contains more live cells as seen in green by SYTO9 staining. These findings indicate that both *copG_1_* and *copG_2_* genes are essential for the symbiotic relationship between SUTN9-2 and legume plants. *copG_1_* is necessary for nodulation, whereas *copG_2_* is crucial for nitrogenase efficiency. Lack of *copG_2_* leads to decreased nitrogenase activity, despite the presence of high nodule numbers. 

### 3.3. The copG_1_ Gene Plays a Crucial Role in the Expression of Nodulation (nod) Genes and Transcriptional Regulator TtsI (ttsI)

**Figure 3:**
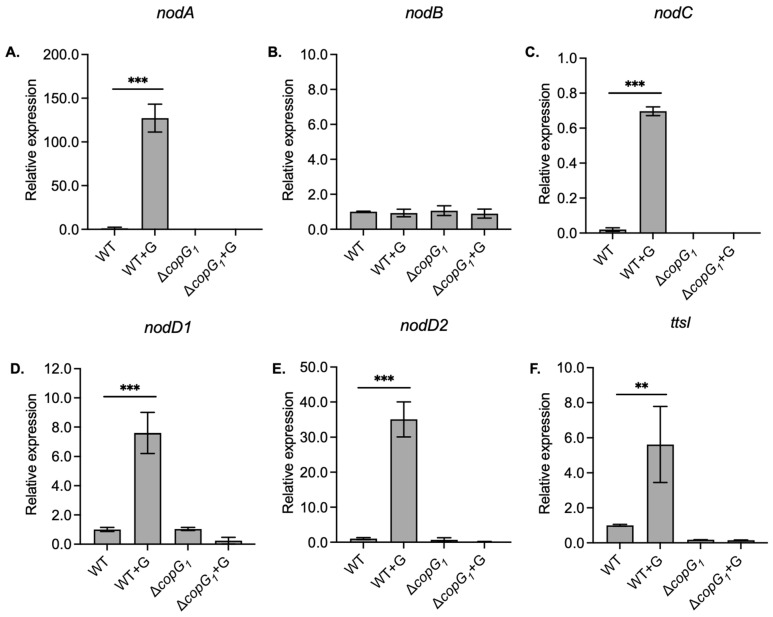
qRT-PCR analysis of *nod* genes from wild-type *Bradyrhizobium* sp. SUTN9-2 (WT) and Δ*copG_1_* grown in the absence and presence of 20 μM genistein (G). Expression of structural genes *nodA* (**A**)*, nodB* (**B**), *nodC* (**C**), regulatory genes *nodD1* (**D**), *nodD2* (**E**), and transcriptional regulator of T3SS, *ttsI* (**F**). The data were normalized in relation to the endogenous control (16S rRNA). Values represent the mean ± SD (*n* = 3). *p* values based on Tukey’s test (** *p* < 0.01, *** *p* < 0.001).

To examine whether *copG_1_* affects the structuring of the NF backbone, transcript levels of *nodABC* and transcriptional activator *nodD* (*nodD1* and *nodD2*) were examined with and without 20 µM genistein induction (Figure 3). The *nodA*, *nodC*, *nodD1*, and *nodD2* genes were almost not expressed in Δ*copG_1_* (Figure 3A,C–E), whereas the expression of *nodB* was not affected by a mutation in *copG_1_* (Figure 3B). The results indicated that *copG_1_* modulates, either directly or indirectly, the expression of *nod* genes, especially the *nodD* gene (Figure 3D,E), which is a transcriptional activator of NFs [43]. Beside NF biosynthesis, NodD1 also activates the transcriptional regulator TtsI (*ttsI*), a gene encoding for T3SS secretion and synthesis [44]. Similar to the *nod* genes, the expression level of *ttsI* was not determined in Δ*copG_1_* in all conditions (Figure 3F). The loss of nodule formation in Δ*copG_1_* may be caused by the suppression of NF synthesis and T3SS due to the absence of *nodD* expression.

### 3.4. Bradyrhizobium sp. SUTN9-2 copG_1_ Is Involved in the Repression of the T4SS Structural Genes traG_1_ and trbE_1_

**Figure 4:**
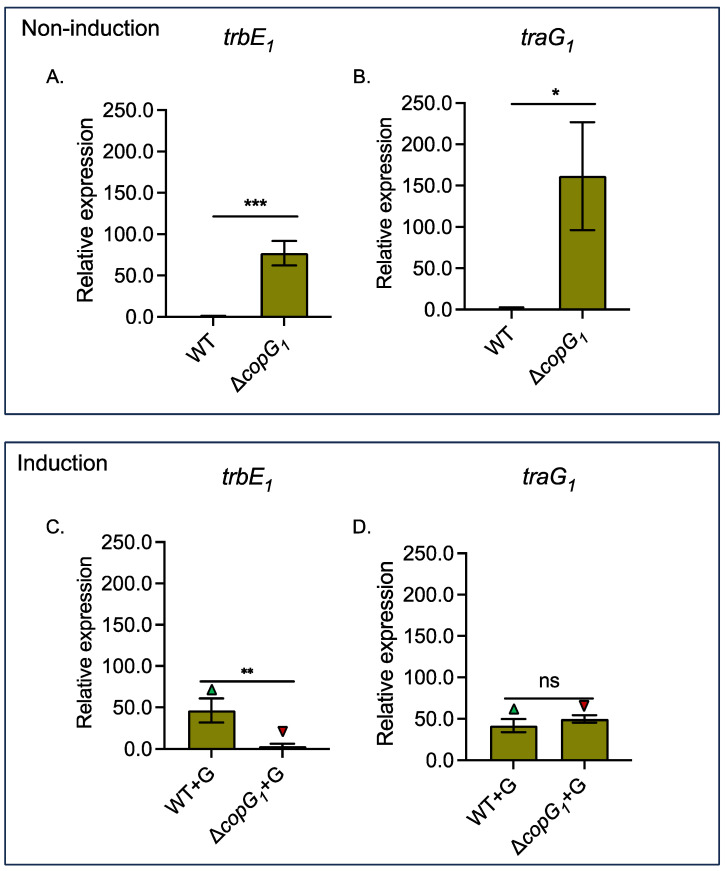
Relative expression of representative T4SS structural genes, including *trbE_1_* (**A**,**C**) and *traG_1_* genes (**B**,**D**) in *Bradyrhizobium* sp. SUTN9-2 (WT) and Δ*copG_1_* with and without 20 μM genistein (G) induction. The 16S rRNA gene was used as an internal control. Values represent the mean ± SD (*n* = 3). *p* values based on the student’s *t*-test (ns *p* > 0.05, * *p* < 0.05, ** *p* < 0.01, *** *p* < 0.001). The green and red arrows represent a statistical increase and decrease, respectively, in gene expression when comparing experiments with and without genistein.

To understand the relationship between the T4SS and *copG_1_* genes more clearly, the gene expression fold changes were examined. T4SS with the *trbE_1_* and *traG_1_* genes showed high expression levels under non-symbiotic conditions when *copG_1_* was deleted (Figure 4A,B). These results indicate that *copG_1_* of SUTN9-2 may act as a suppressor of *trbE_1_* and *traG_1_* under non-symbiotic conditions. However, under mimicked symbiotic conditions with 20 µM genistein induction, the expression of the *trbE_1_* gene significantly decreased in Δ*copG_1_* compared to the wild-type. The expression of the *traG_1_* gene did not differ between the wild-type and the Δ*copG_1_* (Figure 4C,D). However, it is noteworthy that under mimicked symbiosis conditions, CopG_1_ can induce the expression of *trbE_1_* and *traG_1_*, resulting in significantly higher levels than those observed under non-induction conditions. These results suggest that *copG_1_* might act as a synergistic regulator of the T4SS gene in SUTN9-2 under flavonoid induction (Figure 4C,D).

### 3.5. Effect of T4SS and copG_1_ on the Secreted Protein Pattern after 48 h of Genistein Induction 

**Figure 5:**
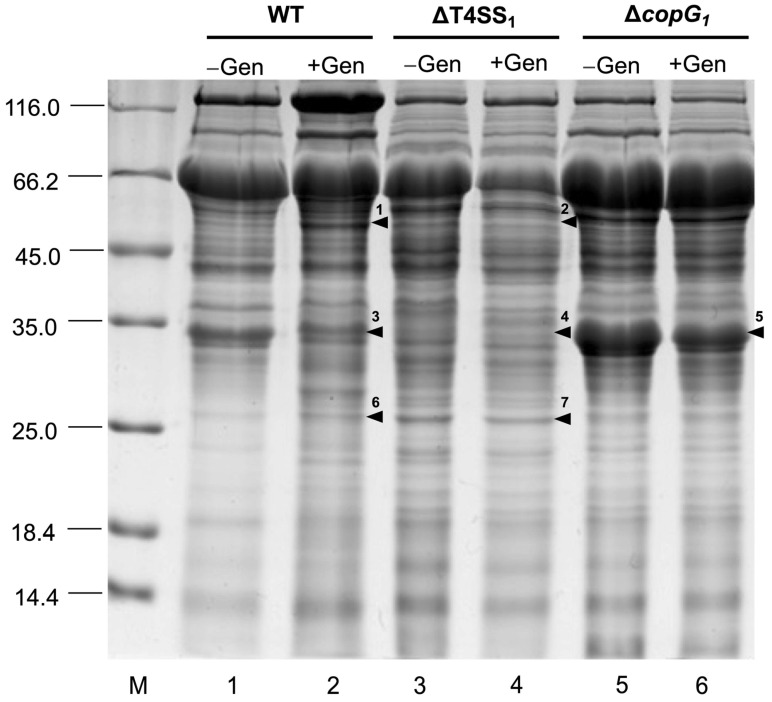
SDA-PAGE analysis of protein secretion into the external medium of *Bradyrhizobium* sp. SUTN9-2 (WT), ΔT4SS_1_, and Δ*copG_1_* with 20 μM genistein (+Gen) and without 20 μM genistein (−Gen) induction. Numbers on the left indicate molecular size markers in kilodaltons. The arrowhead indicates bands identified by mass spectrometry (MS) analysis of WT (1, 3, 6), ΔT4SS_1_ (2, 4, 7), and Δ*copG_1_* (5). (The original SDS-PAGE image was included in the Supplementary Materials as Figure S9).

The secreted protein patterns of *Bradyrhizobium* sp. SUTN9-2, ∆T4SS_1_, and ∆*copG_1_* with 20 µM genistein after 48 h of induction were analyzed by SDS-PAGE. The results revealed distinct protein band patterns under the different conditions (Figure 5). The amino acid sequences of each selected band were examined using mass spectrometry (MS) with the MASCOT program (Table S3 and Figures S5–S8). In the wild-type, genistein induction (band 1) contained a protein matched with the T3SS translocon protein NopX (27%), whereas this band was absent at the same position as ∆T4SS_1_. Similarly, a protein band was observed in the wild-type with genistein induction (band 6), and a match was found with the T3SS host specificity protein NopP (18%). This protein was not observed in ∆T4SS_1_ (band 7) at the same position. While protein bands from the wild-type (band 3) and ∆T4SS_1_ (band 4) were found to be proteins matched with Dct; C_4_-dicarboxylate ABC transporter (31%), it was not observed in ∆*copG_1_* (band 5). Nevertheless, ∆*copG_1_* (band 5) was associated with the amino acid ABC transporter substrate-binding protein glutamate/aspartate transporter subunit (38%). 

**Figure 6:**
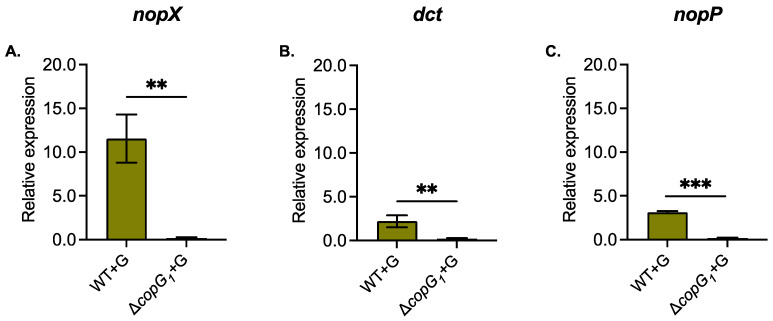
Relative expression of Nodulation outer protein X (*nopX*) (**A**), C_4_-dicarboxylate transporter (*dct*) (**B**), and *nopP* genes (**C**) in *Bradyhizobium* sp. SUTN9-2 (WT) and Δ*copG_1_* under 20 μM genistein induction (+G). The data were normalized in relation to the endogenous control (16S rRNA). Values represent the mean ± SD (*n* = 3). *p* values based on the student’s *t*-test (** *p* < 0.01, *** *p* < 0.001).

qRT-PCR was performed to identify the gene expression of T3SS (*nopP* and *nopX*) and C_4_-dicarboxylate transporter (*dct*), which was not in the secreted protein from ∆*copG_1_*. The results showed that the expression of T3Es (*nopP* and *nopX*) and the C_4_-dicarboxylate transporter (*dct*) under genistein induction was down-regulated in ∆*copG_1_* (Figure 6). This indicated that these genes required *copG_1_* to mediate the regulation under genistein induction. 

## 4. Discussion

At an early nodulation stage of *V. radiata* cv. SUT4, Δ*traG_1_* and Δ*virD2_1_* generated a high number of nodules with smaller sizes compared with the wild-type (Figure 1I,M,Q,R). The symbiosome space of Δ*traG_1_* and Δ*virD2_1_* infecting nodules revealed some dead cells that were not found in the wild-type under confocal microscopy (Figure 1J,N). According to these findings, T4SS is beneficial in the early stages of symbiotic interactions between SUTN9-2 and legumes. Bradyrhizobia have a TraG/Trb operon on the chromosome in the symbiosis island that is similar to that of mesorhizobia based on the *traG* gene’s phylogenetic and gene organization [24]. Beside the structural protein, various bacteria containing T4SS also identified ATPase/Coupling protein, VirD4/TraG, and relaxase VirD2 [45]. The *traG* is commonly found in conjugative plasmids that are responsible for horizontal gene transfer between bacteria. The *traG* gene required to encode the T4SS component served as an ATPase to generate energy during secretion [46]. In addition, TraG also acts as a substrate receptor of T4SS called coupling protein, a substrate receptor that mediates the substrate such as effector proteins, DNA, or a DNA-protein complex through the T4SS channel [16,47,48,49]. In the Pfam prediction, the TraG protein was matched with the Pfam family T4SS-DNA_transfer (PF02534), TrwB_AAD_bound (PF10412), and TraG-D_C (PF12696) (Figure S8A). The C-terminal of this protein can interact with the relaxosome, which is essential for DNA transfer and conjugation in bacteria [46,47,48]. In mesorhizobia, *traG* plays an important role in the early stage of infection, and its expression was observed during induction with root exudate and early nodules generated by *M. mediterraneum* Ca36^T^. Corresponding to mesorhizobia, *traG_1_* of SUTN9-2 may play a crucial role in the initiation of symbiotic interaction with legumes [49]. In *Agrobacterium*, the VirD2 protein is a part of the relaxase family that plays a crucial role in conjugating and mobilizing plasmids that are required for translocation and integration of T-strands into recipient plant cells [50,51]. The conjugative transfer of ICEMlSymR7A in *M. loti* R7A requires VirD2 relaxase to initiate rolling-circle replication [52]. VirD2_1_ of SUTN9-2 possesses a domain of unknown function (DUF), DUF3363, which is an uncharacterized protein (Figure S8B). Although Δ*virD2_1_* had no effect on the number of nodules in *V. radiata* cv. SUT4, nitrogen fixation activity was reduced (Figure 1S,T).

Nodules generated by Δ*virD2_1_* showed many uninfected cells (Figure 1N,P). This finding showed that the communication between SUTN9-2 and the legume at the beginning of nodule organogenesis plays an important role in enhancing infection efficiency and nitrogenase activity after infection. These results strongly indicate that the *traG_1_* and *virD2_1_* genes may be necessary for symbiotic interaction during the early infection stage. Unlike Δ*traG_1_* and Δ*virD2_1_*, Δ*copG_1_* has an impact on the symbiotic interaction between SUTN9-2 and legumes because this mutant was unable to generate nodules with the tested plant (Figure 1E,G). For that reason, *copG_1_* was located downstream of *traG_1_* and *virD2_1_* within the same cluster, it is assumed that *copG_1_* shares a common promoter with *traG_1_* and *virD2_1_*. This observation was supported by a previous study in which T4SS complementation successfully restored nodule formation [24]. Several bacteria, such as *Pseudomonas aeruginosa* [53], *Streptococcus agalactiae* [54], *Vibrio cholerae* [55], *Bradyrhizobium* sp., and *Mesorhizobium* sp., contain the *copG* gene in their genomes [24]. This gene encodes CopG protein, a small transcriptional repressor containing a helix-turn-helix motif domain, which is similar to that of regulatory repressors such as Mnt, Arc, and MetJ in *Salmonella typhimurium* bacteriophage P22 and *Escherichia coli* [45,56,57]. The CopG protein was first discovered in the streptococcal plasmid pMV158 as a transcriptional repressor that interacts with RepB to control the copy number of the plasmid [44,46,58]. In addition, copper resistance was also demonstrated to be influenced by CopG in *P. aeruginosa* and *V. cholerae* [53,55]. 

In SUTN9-2, the CopG_1_ protein was classified as an uncharacterized conserved protein, whereas CopG_2_ was annotated as the Pfam;RHH_1 domain (PF01402), which may serve as a transcriptional regulator within the CopG family (Figure S4) [59]. The removal of both *copG* genes from SUTN9-2 resulted in distinct nodulation efficiency. Even without a nodule generated by Δ*copG_1_*, it can still infect plants because we can monitor both live and dead cells within plant tissues. Surprisingly, live cells were found mostly in the vascular bundle tissue, which is similar to the way of endophytic bacteria behave. These findings imply that *copG_1_* may be crucial for SUTN9-2 in protecting the survival of bacterial cells in the host plant. Bacteria can evolve and adapt to their environment through horizontal gene transfer, usually facilitated by conjugation. Conjugation is a significant biological process because it is the primary way to spread antibiotic resistance genes [60]. Integrative and conjugative elements (ICEs) are another essential mechanism that contributes to conjugation. ICEs are recognized as elements encoded for excision and transferred by conjugation and integration, regardless of the specific mechanisms involved [61]. The T4SS found in SUTN9-2 is classified as a *tra/trb* operon and is recognized for its crucial role in facilitating conjugal transfer. Although SUTN9-2 lacks a conjugation plasmid, ICE is still present on the chromosome. The genes encoding the T4SS_1_ cluster are presented in this ICE, which is an alternative mechanism of genetic exchange in this bacterial strain [24]. To study the impaired nodulation phenotype of Δ*copG_1_* in *V. radiata* cv. SUT4, we analyzed the expression of *nod* genes with and without genistein induction. Common *nod* genes in SUTN9-2, including *nodA* and *nodC* genes, were not expressed even with a lack of *copG_1_* under the flavonoid induction condition, but this did not affect *nodB* expression. In addition, *copG_1_* acts as a stimulator for *nodD1* and *nodD2*, which are the primary transcription factors responsible for NF production. In addition to *nod* genes, the expression level of *ttsI* was not determined in Δ*copG_1_*. The TtsI protein is a transcriptional regulator (previously called *y4xI*) that is activated by flavonoids and NodD1 that bind to conserved sequences called *tts-boxes* [44,56,62]. These proteins are predominantly expressed during the initial infection stages and within mature nodules, and they play a crucial role in enhancing nodulation [57]. During symbiotic interaction, SUTN9-2 required *copG_1_* to mediate the expression of *nod* genes and *ttsI* under flavonoid induction. These results indicate that CopG_1_ may positively mediate the expression of *nod* genes via NodD activation before stimulating NF production, nodule organogenesis, and T3SS. Furthermore, *copG_1_* plays a role as a repressor in T4SS gene expression, suppressing *trbE* and *traG* expression under flavonoid stimulation (Figure 4C,D). In contrast, these genes were not affected by flavonoids in the absence of the *copG_1_* gene. 

The protein expression profiles of SUTN9-2, ∆T4SS_1,_ and ∆*copG_1_* with genistein treatment were analyzed by SDS-PAGE. The results revealed distinct protein band patterns under different conditions (Figure 5). ∆*copG_1_* exhibited a deficiency in producing nodules in various plant species and a striking increase in protein expression compared with the wild-type. According to the analysis of the *copG_1_* domain protein (Figure S4), *CopG_1_* was predicted to be a transcriptional regulator that might play a role in the regulation of gene expression. The Δ*copG_1_* lane appears to have much more protein intensity overall because the proportion of protein in this lane might be less than in other lanes. Therefore, 10 µg might show a higher band intensity. A comparative proteomic analysis of the whole secretome should be conducted further to identify additional target proteins involved in this interaction. It was found that several proteins were secreted, but the C_4_-dicarboxylate transport system (*dct*) protein was not identified in ∆*copG_1_*, and this result corresponded to the downregulation of the *dct* gene quantified by qRT-PCR (Figure 6). The *dct* gene plays a crucial role in symbiosis numerous rhizobia [58]. For example, the *dct* mutant of *S. meliloti* and *R. trifolii* can generate ineffective nodules with the host legume [58,63]. In addition to the *dct* gene, other genes are expressed in the same pattern, including *nopX* and *nopP,* which are also essential for symbiosis (Figure 6). NopX is a component of T3SS as a translocation pore (translocon) apparatus that is important for host-specific interaction between the rhizobium and host plant. The NGRΔ*nopX* has a significant effect on nodule number because this mutant forms fewer nodules in all plant species tested, including *Flemingia congesta*, *Tephrosia vogelii*, *Pachyrhizus tuberosus,* and *Lablab purpureus* [64]. NopP is a T3SS-effector protein that is phosphorylated by plant kinases [65]. Lack of NopP in *Rhizobium* sp. NGR234 reduces the capacity of nodule organogenesis in tropical legumes. This indicates a positive effect of NopP on symbiosis [64]. NopP of *B. diazoefficiens* USDA122 is necessary and causes Rj2-dependent incompatibility [66]. The T3SS of SUTN9-2 has no impact on its symbiotic relationship with *V. radiata* [18]. However, based on the protein secretion results of T3Es (NopP and NopX) and *nodD* gene expression, it is evident that *copG_1_* regulates the function of *nodD* and T3SS. Previous reports indicated that *nodD* controls the function of the *nod* cluster by binding to the *nod box* region. Similarly, *nodD* can regulate T3SS function by binding to *ttsI* [62]. Therefore, the results of this experiment confirm that CopG_1_ controls the function of *nodD*, influencing the expression of *nod* cluster genes and T3SS. Perhaps CopG_1_ is a crucial factor in the early stages of legume and SUTN9-2 communication. It is plausible that the regulatory system governing the expression of *nod* genes does not solely depend on the interaction between flavonoids and NodD. Another factor, CopG_1_, also collaborates with flavonoids and NodD to regulate the expression of *nod* genes and T3SS. Carbon and nitrogen metabolism are the primary mechanisms necessary for the exchange of nutrients between plant and bacterial partners. The proteins secreted from ∆*copG_1_* matched the periplasmic binding proteins of the glutamate/aspartate ABC transporter. Glutamate is a significant contributor to the total metabolite content, which plays an essential role in nitrogen metabolism, amino acid metabolism, transamination, and carbon sources [67,68]. During symbiosis, the main carbon source utilized by rhizobia is C_4_-dicarboxylic acid [69,70]. In the mimicked symbiotic conditions, ∆*copG_1_* lost the ability to establish a symbiotic interaction. Thereafter, increasing the glutamate/aspartate ABC transporter may promote carbon and nitrogen uptake to support bacterial cell survival, but it is not necessary for symbiotic interaction. This again suggests that *copG_1_* may act as a regulator of *nodD* and T4SS gene expression under symbiotic conditions. The deeper insights into the genetic mechanisms of T4SS genes involved in rhizobia and legume symbiosis should be further investigated through the transcriptomics analysis to obtain the comprehensive gene expression profiles as well as may explore gene expression on the host side for more understanding.

## 5. Conclusions

This is the first report about genes related T4SS in *Bradyrhizobium* sp. SUTN9-2 that involved in the symbiosis interaction with legumes. This finding reveals that T4SS_1_ containing *copG_1_*, *traG_1_*, and *virD2_1_* has beneficial effects on symbiotic interactions with diverse legumes. The early stage of infection and nodulation is influenced by *traG_1_* and *virD2_1_*. While, *copG_1_* is necessary for nodulation, the essential role of *copG_2_* is nitrogen fixation efficiency. Additionally, *copG_1_* served as a suppressor of T4SS genes under non-induction conditions and was required to stimulate the expression of T4SS genes through flavonoid induction. *copG_1_* also acted as a suppressor of secreted protein under flavonoid induction conditions. In addition, a lack of *copG_1_* led to suppressed expression of *nopX*, *nopP*, and *dct*, which are important for infection and nodulation during symbiosis. Thus, *copG_1_* is most likely responsible for regulation via functions in T3SS, *nodD* regulation, and the carbon and nitrogen exchange systems, which are significant for SUTN9-2 during symbiosis. In this study, *copG_1_* was discovered as a new transcriptional factor in the T4SS cluster and is important for host specificity and competition during symbiosis with their host. Knowledge from this research serves as a model for studying the interaction between the host plant and the secretion systems of *Bradyrhizobium* that further facilitates scientists to identify effectors required for better colonization, enhancing nodulation and nitrogen fixation, which lead to an increase in legume crop yields under sustainable agriculture.

## Figures and Tables

**Table 1:** Primers used in this study.

Name	Sequences (5′-3′)	Descriptions
Primers for gene deletion		
Up.*copG_1_*. *XbaI*.F	CCT TGA GAT CTA GAT GTA GTC TGC CCC GAA GTA GC	These primer sets were used to obtain the deletion of the *copG_1_* gene of *Bradyrhizobium* sp. SUTN9-2 by double crossing over.
Up. *copG_1_*. overl. *HindIII*. R	GAG GCG GAC ATG AAA GCT TAA TGA AGG CGG ACG GCC ACT AG	
Dw. *copG_1_*. overl. *HindIII.* F	GTC CGC CTT CAT TAA GCT TTC ATG TCC GCC TCA CAG TCC GA	
Dw. *copG_1_*. *EcoRI*.R	AGA TCG GGA ATT CGT TGA CCG AGG ATC TTC AGG CCA	
Up. *copG_2_*. *XbaI*.F	GCC GTT TCT AGA ATT GCG ACA ACG GAC CAG GGC AA	These primer sets were used to obtain the deletion of the *copG_2_* gene of *Bradyrhizobium* sp. SUTN9-2 by double crossing over.
Up. *copG_2_*. overl. *HindIII.* R	GCG CGA CCG AAT GAA GCT TAA GCT GGT CAC GCT ATC GGC T	
Dw. *copG_2_*. overl. *HindIII*. F	GCG TGA CCA GCT TAA GCT TCA TTC GGT CGC GCA TAT TGC C	
Dw. *copG_2_*. *EcoRI*. R	CTG TCC GAA TTC ATG TCG TTC CTC GGG TTG TAC C	
Up. *traG_1_*. *XbaI.* F	TTC GGG TCT AGA TGT AGT CTG CCC CGA AGT AGC	These primer sets were used to obtain the deletion of the *traG_1_* gene of *Bradyrhizobium* sp. SUTN9-2 by double crossing over.
Up. *traG_1_*. overl. *BamHI*	TCC CTC CAA TCA CGG ATC CAT CCT GGT GAC GAT CTC GGA C	
Dw. *traG_1_*. overl. *BamHI*	TCG TCA CCA GGA TGG ATC CGT GAT TGG AGG GAT CGT TCA CAG	
Dw. *traG_1_*. *EcoRI.*R	CCG GCT GAA TTC CTT GGA AAG CCT TGG TCT CG	
Up. *virD2_1_*. XbaI. F	ACC GGC TTC TAG AAG ATG CGC AGT CCG CAT CAT C	These primer sets were used to obtain the deletion of the *virD2_1_* gene of *Bradyrhizobium* sp. SUTN9-2 by double crossing over.
Up. *virD2_1_*. overl. *BamHI*	GAG GAG AAG GAA TGG ATC CTG AAC GAT CCC TCC AAT CAC CG	
Dw. *virD2_1_*. overl. *BamHI*	GAG GGA TCG TTC AGG ATC CAT TCC TTC TCC TCA GCC ATG GC	
Dw. *virD2_1_*. *EcoRI.* R	CCA TCG GAA TTC TTG TCG ATG CGG AGG AGG CAT C	
Primers for qRT-PCR analysis		
SUTN9-2. *nodA*. F	GTT CAA TGC GCA GCC CTT TGA G	Specific primers for *nodA* gene expression in SUTN9-2 on the chromosome
SUTN9-2. *nodA*. R	ATT CCG AGT CCT TCG AGA TCC G	
SUTN9-2. *nodC*. F	ATT GGC TCG CGT GCA ACG AAG A	Specific primers for *nodC* gene expression in SUTN9-2 on the chromosome
SUTN9-2. *nodC*. R	AAT CAC TCG GCT TCC CAC GGA A	
SUTN9-2. *nodD1*. F	ATT CGT CTC CTC AGA CCG TGC T	Specific primers for *nodD1* gene expression in SUTN9-2 on the chromosome
SUTN9-2. *nodD1*. R	TTC ATG TCG AGT GCG CAC CCT A	
SUTN9-2. *nodD2*. F	TGC TTA ACT GCA ACG TGA CCC	Specific primers for *nodD2* gene expression in SUTN9-2 on the chromosome
SUTN9-2. *nodD2*. R	ATG AGC ACG AGG AGC TTC TC	
SUTN9-2. *trbE_1_*. F	GAT TGC AGG AGA ACC GTG AGG C	Specific primers for *trbE_1_* gene expression in SUTN9-2 on the chromosome
SUTN9-2. *trbE_1_*. R	AAC AGC GCC GAG GAT TCA GTC T	
SUTN9-2. *traG_1_*. F	TTC TCG ATC TGG TTC AGC GAC TG	Specific primers for *traG_1_* gene expression in SUTN9-2 on the chromosome
SUTN9-2. *traG_1_*. R	TTG ACC GAG GAT CTT CAG GCC A	
SUTN9-2. *ttsI*. F	ATG AGT TCG TCG GTG GAC AC	Specific primers for transcriptional regulator TtsI (*ttsI*) gene expression in SUTN9-2 on chromosome
SUTN9-2. *ttsI*. R	CCA CAT GGT CCT GCT CGA AT	
16s. F	ATT ACC GCG GCT GCT GG	Universal primers for 16S rRNA are used as an internal control for bacterial gene expression [31]
16s. R	ACT CCT ACG CGA GGC AGC AG	
*dct*. F	CGA CTA TCA GGG CGT GAA AT	Specific primers for C_4_-dicarboxylate transport (*dct*) gene expression in SUTN9-2 on chromosome
*dct*. R	TCC AGC AAT CAG ACC TGT G	
*nopX*. F	GGGTGGTCGAGGAAGTATTG	Specific primers for Type III secretion system (T3SS) gene expression in SUTN9-2 on chromosome
*nopX*. R	GGTTATGACCCAGACCGATG	
*nopP*. F	GGTCACACCGACGAAGATAC	
*nopP*. R	CCGAAGATCCACTTGGGATG	

## Data Availability

All the required data are available in the manuscript and Supplementary Materials.

## Supplementary Materials

The following supporting information can be downloaded at: https://www.mdpi.com/article/10.3390/biology13060415/s1. Figure S1. Genetic organization of type IV secretion system cluster 1 (T4SS_1_) and cluster 2 (T4SS_2_) on the chromosome of *Bradyrhizobium* sp. SUTN9-2; Figure S2. CLUSTALW (2.1) Multiple sequence alignments of *copG_1_* and *copG_2_* gene sequences in *Bradyrhizobium* sp. SUTN9-2; Figure S3. CLUSTALW (2.1) Multiple sequence alignments of CopG_1_ and CopG_2_ amino acid sequences in *Bradyrhizobium* sp. SUTN9-2; Figure S4. Protein domain architecture analysis of CopG proteins in bradyrhizobia; Figure S5. Amino acid sequences of protein selected band 1; Figure S6. Amino acid sequences of protein selected band 3; Figure S7. Amino acid sequences of protein selected band 6; Figure S8. Protein domain architecture analysis of TraG_1_ (A) and VirD2_1_ (B) protein sequences in *Bradyrhizobium* sp. SUTN9-2; Figure S9. Original SDS-PAGE of proteins secretion into the external medium of *Bradyrhizobium* sp. SUTN9-2 (WT) and mutant strains with 20 μM genistein (+Gen) and without 20 μM genistein (−Gen) induction. Table S1. Nodulation pouch test inoculated with *Bradyrhizobium* sp. SUTN9-2 and ∆*copG_1_* in various legumes; Table S2. Bacterial strains and plasmids used in this study; Table S3. Identification and characterization of selected protein bands from *Bradyrhizobium* sp. SUTN9-2 with genistein induction.

## References

[1] Goyal R.K., Habtewold J.Z. (2023). Evaluation of legume–rhizobial symbiotic interactions beyond nitrogen fixation that help the host survival and diversification in hostile environments. Microorganisms.

[2] Rascio N., La Rocca N. (2013). Biological Nitrogen Fixation. Reference Module in Earth Systems and Environmental Sciences.

[3] Granada Agudelo M., Ruiz B., Capela D., Remigi P. (2023). The role of microbial interactions on rhizobial fitness. Front. Plant Sci..

[4] Dénarié J., Debellé F., Promé J.-C. (1996). *Rhizobium* lipo-chitooligosaccharide nodulation factors: Signaling molecules mediating recognition and morphogenesis. Annu. Rev. Biochem..

[5] Green E.R., Mecsas J. (2016). Bacterial secretion systems: An overview. Microbiol. Spectr..

[6] Gassmann W., Bhattacharjee S. (2012). Effector-triggered immunity signaling: From gene-for-gene pathways to protein-protein interaction networks. Mol. Plant-Microbe Interact..

[7] Jones J.D.G., Dangl J.L. (2006). The plant immune system. Nature.

[8] Fauvart M., Michiels J. (2008). Rhizobial secreted proteins as determinants of host specificity in the *Rhizobium*–legume symbiosis. FEMS Microbiol. Lett..

[9] Nelson M.S., Sadowsky M.J. (2015). Secretion systems and signal exchange between nitrogen-fixing rhizobia and legumes. Front. Plant Sci..

[10] Zboralski A., Biessy A., Filion M. (2022). Bridging the gap: Type III secretion systems in plant-beneficial bacteria. Microorganisms.

[11] Luo L., Lu D. (2014). Immunosuppression during *Rhizobium* -legume symbiosis. Plant Signal. Behav..

[12] Jiménez-Guerrero I., Medina C., Vinardell J.M., Ollero F.J., López-Baena F.J. (2022). The rhizobial type 3 secretion system: The Dr. Jekyll and Mr. Hyde in the *Rhizobium*–legume symbiosis. Int. J. Mol. Sci..

[13] Songwattana P., Chaintreuil C., Wongdee J., Teulet A., Mbaye M., Piromyou P., Gully D., Fardoux J., Zoumman A.M.A., Camuel A. (2021). Identification of type III effectors modulating the symbiotic properties of *Bradyrhizobium vignae* strain ORS3257 with various *Vigna* species. Sci. Rep..

[14] Salinero-Lanzarote A., Pacheco-Moreno A., Domingo-Serrano L., Durán D., Ormeño-Orrillo E., Martínez-Romero E., Albareda M., Palacios J.M., Rey L. (2019). The type VI secretion system of *Rhizobium etli* Mim1 has a positive effect in symbiosis. FEMS Microbiol. Ecol..

[15] Tighilt L., Boulila F., De Sousa B.F.S., Giraud E., Ruiz-Argüeso T., Palacios J.M., Imperial J., Rey L. (2022). The *Bradyrhizobium* sp. LmicA16 type VI secretion system is required for efficient nodulation of *Lupinus* spp.. Microb. Ecol..

[16] Fronzes R., Christie P.J., Waksman G. (2009). The structural biology of type IV secretion systems. Nat. Rev. Microbiol..

[17] Smith E.F., Townsend C.O. (1907). A plant-tumor of bacterial origin. Science.

[18] Piromyou P., Songwattana P., Teamtisong K., Tittabutr P., Boonkerd N., Tantasawat P.A., Giraud E., Göttfert M., Teaumroong N. (2019). Mutualistic co-evolution of T3SSs during the establishment of symbiotic relationships between *Vigna radiata* and bradyrhizobia. MicrobiologyOpen.

[19] Kaneko T., Maita H., Hirakawa H., Uchiike N., Minamisawa K., Watanabe A., Sato S. (2011). Complete genome sequence of the soybean symbiont *Bradyrhizobium japonicum* strain USDA6^T^. Genes.

[20] Cytryn E.J., Jitacksorn S., Giraud E., Sadowsky M.J. (2008). Insights learned from pBTAi1, a 229-Kb accessory plasmid from *Bradyrhizobium* sp. strain BTAi1 and prevalence of accessory plasmids in other *Bradyrhizobium* sp. strains. ISME J..

[21] Okazaki S., Noisangiam R., Okubo T., Kaneko T., Oshima K., Hattori M., Teamtisong K., Songwattana P., Tittabutr P., Boonkerd N. (2015). Genome analysis of a Novel *Bradyrhizobium* sp. DOA9 carrying a symbiotic plasmid. PLoS ONE.

[22] Piromyou P., Songwattana P., Greetatorn T., Okubo T., Kakizaki K.C., Prakamhang J., Tittabutr P., Boonkerd N., Teaumroong N., Minamisawa K. (2015). The type III secretion system (T3SS) is a determinant for rice-endophyte colonization by non-photosynthetic *Bradyrhizobium*. Microbes Environ..

[23] Greetatorn T., Hashimoto S., Sarapat S., Tittabutr P., Boonkerd N., Uchiumi T., Teaumroong N. (2019). Empowering rice seedling growth by endophytic *Bradyrhizobium* sp. SUTN 9-2. Lett. Appl. Microbiol..

[24] Wangthaisong P., Piromyou P., Songwattana P., Wongdee J., Teamtaisong K., Tittabutr P., Boonkerd N., Teaumroong N. (2023). The type IV secretion system (T4SS) mediates symbiosis between *Bradyrhizobium* sp. SUTN9-2 and legumes. Appl. Environ. Microbiol..

[25] Zhang Y.F., Wang E.T., Tian C.F., Wang F.Q., Han L.L., Chen W.F., Chen W.X. (2008). *Bradyrhizobium elkanii*, *Bradyrhizobium yuanmingense* and *Bradyrhizobium japonicum* are the main rhizobia associated with *Vigna unguiculata* and *Vigna radiata* in the subtropical region of China. FEMS Microbiol. Lett..

[26] Albareda M., Rodríguez-Navarro D.N., Temprano F.J. (2009). Soybean inoculation: Dose, N fertilizer supplementation and rhizobia persistence in soil. Field Crops Res..

[27] Sadowsky M.J., Tully R.E., Cregan P.B., Keyser H.H. (1987). Genetic diversity in *Bradyrhizobium Japonicum* serogroup 123 and its relation to genotype-specific nodulation of soybean. Appl. Environ. Microbiol..

[28] Tsai J.-W., Alley M.R.K. (2000). Proteolysis of the McpA chemoreceptor does not require the *Caulobacter* major chemotaxis operon. J. Bacteriol..

[29] Blondelet-Rouault M.-H., Weiser J., Lebrihi A., Branny P., Pernodet J.-L. (1997). Antibiotic resistance gene cassettes derived from the π interposon for use in *E. coli* and *Streptomyces*. Gene.

[30] Ditta G., Stanfield S., Corbin D., Helinski D.R. (1980). Broad host range DNA cloning system for Gram-negative bacteria: Construction of a gene bank of *Rhizobium meliloti*. Proc. Natl. Acad. Sci. USA.

[31] Muyzer G., De Waal E.C., Uitterlinden A.G. (1993). Profiling of complex microbial populations by denaturing gradient gel electrophoresis analysis of polymerase chain reaction-amplified genes coding for 16S rRNA. Appl. Environ. Microbiol..

[32] Teamtisong K., Songwattana P., Noisangiam R., Piromyou P., Boonkerd N., Tittabutr P., Minamisawa K., Nantagij A., Okazaki S., Abe M. (2014). Divergent *nod*-containing *Bradyrhizobium* sp. DOA9 with a megaplasmid and its host range. Microbes Environ..

[33] Ehrhardt D., Atkinson E. (1992). Long depolarization of Alfalfa root hair membrane potential by *Rhizobium meliloti* Nod factors. Science.

[34] Renier S., Hébraud M., Desvaux M. (2011). Molecular biology of surface colonization by *Listeria monocytogenes*: An additional facet of an opportunistic Gram-positive Foodborne Pathogen. Environ. Microbiol..

[35] Somasegaran P., Hoben H.J. (1994). Handbook for Rhizobia.

[36] Phimphong T., Sibounnavong P., Phommalath S., Wongdee J., Songwattana P., Piromyou P., Greetatorn T., Boonkerd N., Tittabutr P., Teaumroong N. (2023). Selection and evaluation of Bradyrhizobium inoculum for Peanut, *Arachis hypogea* production in the Lao People’s Democratic Republic. J. Appl. Nat. Sci..

[37] Bradford M. (1976). A rapid and sensitive method for the quantitation of microgram quantities of protein utilizing the principle of protein-dye binding. Anal. Biochem..

[38] Laemmli U.K. (1970). Cleavage of structural proteins during the assembly of the head of Bacteriophage T4. Nature.

[39] Haag A.F., Baloban M., Sani M., Kerscher B., Pierre O., Farkas A., Longhi R., Boncompagni E., Hérouart D., Dall’Angelo S. (2011). Protection of *Sinorhizobium* against host cysteine-rich antimicrobial peptides is critical for symbiosis. PLoS Biol..

[40] Vallenet D., Calteau A., Dubois M., Amours P., Bazin A., Beuvin M., Burlot L., Bussell X., Fouteau S., Gautreau G. (2019). MicroScope: An integrated platform for the annotation and exploration of microbial gene functions through genomic, pangenomic and metabolic comparative analysis. Nucleic Acids Res..

[41] Letunic I., Khedkar S., Bork P. (2021). SMART: Recent updates, New developments and status in 2020. Nucleic Acids Res..

[42] Paysan-Lafosse T., Blum M., Chuguransky S., Grego T., Pinto B.L., Salazar G.A., Bileschi M.L., Bork P., Bridge A., Colwell L. (2023). InterPro in 2022. Nucleic Acids Res..

[43] Heidstra R., Bisseling T. (1996). Nod factor-induced host responses and mechanisms of Nod factor perception. New Phytol..

[44] Marie C., Deakin W.J., Ojanen-Reuhs T., Diallo E., Reuhs B., Broughton W.J., Perret X. (2004). TtsI, a key regulator of *Rhizobium* species NGR234 is required for type III-dependent protein secretion and synthesis of rhamnose-rich polysaccharides. Mol. Plant-Microbe Interact..

[45] Tegtmeyer N., Linz B., Yamaoka Y., Backert S. (2022). Unique TLR9 activation by *Helicobacter pylori* depends on the *cag* T4SS, but not on VirD2 relaxases or VirD4 coupling proteins. Curr. Microbiol..

[46] Schröder G., Krause S., Zechner E.L., Traxler B., Yeo H.-J., Lurz R., Waksman G., Lanka E. (2002). TraG-like proteins of DNA transfer systems and of the *Helicobacter pylori* ype IV secretion system: Inner membrane gate for exported substrates?. J. Bacteriol..

[47] Gunton J.E., Gilmour M.W., Baptista K.P., Lawley T.D., Taylor D.E. (2007). Interaction between the co-inherited TraG coupling protein and the TraJ membrane-associated protein of the H-plasmid conjugative DNA transfer system resembles chromosomal DNA translocases. Microbiology.

[48] Lu J., Wong J.J.W., Edwards R.A., Manchak J., Frost L.S., Glover J.N.M. (2008). Structural basis of specific TraD-TraM recognition during F plasmid-mediated bacterial conjugation. Mol. Microbiol..

[49] Paço A., Da-Silva J.R., Eliziário F., Brígido C., Oliveira S., Alexandre A. (2019). TraG Gene Is Conserved across Mesorhizobium Spp. Able to Nodulate the Same Host Plant and Expressed in Response to Root Exudates. BioMed Res. Int..

[50] Byrd D.R., Matson S.W. (1997). Nicking by transesterification: The reaction catalysed by a relaxase. Mol. Microbiol..

[51] Van Kregten M., Lindhout B.I., Hooykaas P.J.J., Van Der Zaal B.J. (2009). *Agrobacterium*-mediated T-DNA transfer and integration by minimal VirD2 consisting of the relaxase domain and a type IV secretion system translocation signal. Mol. Plant-Microbe Interact..

[52] Ramsay J.P., Sullivan J.T., Stuart G.S., Lamont I.L., Ronson C.W. (2006). Excision and Transfer of the *Mesorhizobium loti* R7A symbiosis island requires an integrase IntS, a novel recombination directionality factor RdfS, and a putative relaxase RlxS. Mol. Microbiol..

[53] Hausrath A.C., Ramirez N.A., Ly A.T., McEvoy M.M. (2020). The bacterial copper resistance protein CopG contains a cysteine-bridged tetranuclear copper cluster. J. Biol. Chem..

[54] Gomis-Ruth F.X. (1998). The structure of plasmid-encoded transcriptional repressor CopG unliganded and bound to its operator. EMBO J..

[55] Marrero K., Sánchez A., González L.J., Ledón T., Rodríguez-Ulloa A., Castellanos-Serra L., Pérez C., Fando R. (2012). Periplasmic proteins encoded by VCA0261–0260 and VC2216 genes together with *copA* and *cueR* products are required for copper tolerance but not for virulence in *Vibrio cholerae*. Microbiology.

[56] Kobayashi H., Graven Y.N., Broughton W.J., Perret X. (2004). Flavonoids induce temporal shifts in gene-expression of *nod*-box controlled loci in *Rhizobium* sp. NGR234. Mol. Microbiol..

[57] Cao Y., Halane M.K., Gassmann W., Stacey G. (2017). The role of plant innate immunity in the legume-*Rhizobium* symbiosis. Annu. Rev. Plant Biol..

[58] Yurgel S.N., Kahn M.L. (2005). *Sinorhizobium meliloti dctA* mutants with partial ability to transport dicarboxylic acids. J. Bacteriol..

[59] Acebo P., García De Lacoba M., Rivas G., Andreu J.M., Espinosa M., Solar G.D. (1998). Structural features of the plasmid pMV158-encoded transcriptional repressor CopG, a protein sharing similarities with both helix-turn-helix and β-sheet DNA binding proteins. Proteins Struct. Funct. Genet..

[60] Guglielmini J., Quintais L., Garcillán-Barcia M.P., de la Cruz F., Rocha E.P.C. (2011). The Repertoire of ICE in prokaryotes underscores the unity, diversity, and ubiquity of conjugation. PLoS Genet..

[61] Bellanger X., Payot S., Leblond-Bourget N., Guédon G. (2014). Conjugative and mobilizable genomic islands in bacteria: Evolution and diversity. FEMS Microbiol. Rev..

[62] Krause A., Doerfel A., Göttfert M. (2002). Mutational and transcriptional analysis of the type III secretion system of *Bradyrhizobium Japonicum*. Mol. Plant-Microbe Interact..

[63] Ronson C.W., Lyttleton P., Robertson J.G. (1981). C_4_-dicarboxylate transport mutants of *Rhizobium trifolii* form ineffective nodules on *Trifolium repens*. Proc. Natl. Acad. Sci. USA.

[64] Marie C., Deakin W.J., Viprey V., Kopciñska J., Golinowski W., Krishnan H.B., Perret X., Broughton W.J. (2003). Characterization of Nops, nodulation outer proteins, secreted via the type III secretion system of NGR234. Mol. Plant-Microbe Interact..

[65] Bartsev A.V., Boukli N.M., Deakin W.J., Staehelin C., Broughton W.J. (2003). Purification and phosphorylation of the effector protein NopL from *Rhizobium* sp. NGR234. FEBS Lett..

[66] Sugawara M., Takahashi S., Umehara Y., Iwano H., Tsurumaru H., Odake H., Suzuki Y., Kondo H., Konno Y., Yamakawa T. (2018). Variation in bradyrhizobial NopP effector determines symbiotic incompatibility with Rj2-soybeans via effector-triggered immunity. Nat. Commun..

[67] Streeter J.G. (1987). Effect of nitrate on the organic acid and amino acid composition of legume nodules. Plant Physiol..

[68] Forde B.G., Lea P.J. (2007). Glutamate in plants: Metabolism, regulation, and signalling. J. Exp. Bot..

[69] Finan T.M., Wood J.M., Jordan D.C. (1983). Symbiotic properties of C_4_-dicarboxylic acid transport mutants of *Rhizobium leguminosarum*. J. Bacteriol..

[70] Jording D., Sharma P.K., Schmidt R., Engelke T., Uhde C., Pühler A. (1993). Regulatory aspects of the C_4_-dicarboxylate transport in *Rhizobium meliloti*: Transcriptional activation and dependence on effectave symbiosis. J. Plant Physiol..

