# Supplementary material for: CopG1, a Novel Transcriptional Regulator Affecting Symbiosis in Bradyrhizobium sp. SUTN9-2
Source: Biology (Basel). 2024 Jun 5;13(6):415. doi: 10.3390/biology13060415 (PMC11201211; doi:10.3390/biology13060415)
Supplement: Supplementary file 1 [file biology-13-00415-s001.zip › biology-3018698-Supplementary Materials.pdf]

Supplementary Materials

Title : $CopG_1$, a novel transcriptional regulator affecting symbiosis in *Bradyrhizobium* sp. SUTN9-2

Praneet Wangthaisong [1], Pongdet Piromyou [2], Pongpan Songwattana [2], Tarnee Phimphong [1], Apisit Songsaeng [1], Natcha Pruksametanan [1], Pakpoom Boonchuen [1], Jenjira Wongdee [2], Kamonluck Teamtaisong [3], Nantakorn Boonkerd [1], Shusei Sato [4], Panlada Tittabutr [1,*], Neung Teaumroong [1,*]

[1] School of Biotechnology, Institute of Agricultural Technology, Suranaree University of Technology, Nakhon Ratchasima 30000, Thailand.

[2] Institute of Research and Development, Suranaree University of Technology, Nakhon Ratchasima 30000, Thailand.

[3] The Center for Scientific and Technological Equipment, Suranaree University of Technology, Nakhon Ratchasima, Thailand

[4] Graduate School of Life Sciences, Tohoku University, Sendai 980-8577, Japan.

Table S1. Bacterial strains and plasmids used in this study

Strain or plasmid	Relevant characteristics	Reference or source
**Strain**		
Bradyrhizobium sp.		
SUTN9-2	*A. americana* nodule isolate (paddy crop)	
Δ$copG_1$	SUTN9-2 derivative containing an Ω cassette insertion at *HindIII* site, *copG* copy 1::sm/sp; Sm^r, Sp^r	This study
Δ$copG_2$	SUTN9-2 derivative containing an Ω cassette insertion at *BamHI* site, *copG* copy 2::sm/sp; Sm^r, Sp^r	This study
Δ$traG_1$	SUTN9-2 derivative containing an Ω cassette insertion at *BamHI* site, *traG* copy 1::sm/sp; Sm^r, Sp^r	This study
Δ$virD2_1$	SUTN9-2 derivative containing an Ω cassette insertion at *BamHI* site, *virD2* copy 1::sm/sp; Sm^r, Sp^r	This study
Escherichia coli		
DH5α	*supE44 ΔlacU169 hsdR17 recA1 endA1 gyrA96 thi-1 relA1*	Toyobo Inc.
**Plasmid**		
pRK2013	ColE1 replicon carrying *RK2* transfer genes; Km^r; Helper plasmid	[1]
pNTPS129	Cloning vector harboring *sacB* gene under the control of the constitutive *npt2* promoter; Km^r	[2]
pNTPS129-Δ$copG_1$	pNTPS129-*npt2*-*sacB* containing the flanking region of *copG* copy 1	This study
pNTPS129-Δ$copG_2$	pNTPS129-*npt2*-*sacB* containing the flanking region of *copG* copy 2	This study
pNTPS129-Δ$traG_1$	pNTPS129-*npt2*-*sacB* containing the flanking region of *traG* copy 1	This study
pNTPS129-Δ$virD2_1$	pNTPS129-*npt2*-*sacB* containing the flanking region of *virD2* copy 1	This study

Table S2. Nodulation pouch test inoculated with *Bradyrhizobium* sp. SUTN9-2 and Δ*copG*$_1$ in various legumes.

**Plants**	**Nodulation** [a]	
	**SUTN9-2**	**Δ*copG*$_1$**
Papilionoideae		
Genistoids		
Crotalaria juncea	(+)	(-)
Dalbergioids		
Aeschynomene americana cv. Thai	(+)	(-)
Arachis hypogaea cv. Thainan-9	(+)	(-)
A. hypogaea cv. Khonkaen 5	(+)	(-)
Millettioids		
Indigofera tinctoria	(+)	(-)
Macroptilium atropurpureum	(+)	(-)
Vigna radiata cv. SUT1	(+)	(-)
V. radiata cv. CN72	(+)	(-)
V. radiata cv. KUML4	(+)	(-)
V. radiata cv. CN36	(+)	(-)
V. radiata cv. KPS1	(+)	(-)
V. mungo cv. U thong 2	(+)	(-)
V. subterranean	(+)	(-)

[a] (+), Normal nodule formation; (-),Cannot form nodules

Table S3. Identification and characterization of selected protein bands from *Bradyrhizobium* sp. SUTN9-2 with genistein induction (bands 1, 3 and 6) using the MASCOT program and NCBI BLAST.

Protein band	Protein name	Sequences coverage (%)	PI	Score	NCBI accession no.
1	NopX	27%	5.12	1283	PWE77101.1
3	Dct	31%	8.86	306	WP_036025066.1
6	NopP	18%	4.88	120	PWE81636.1

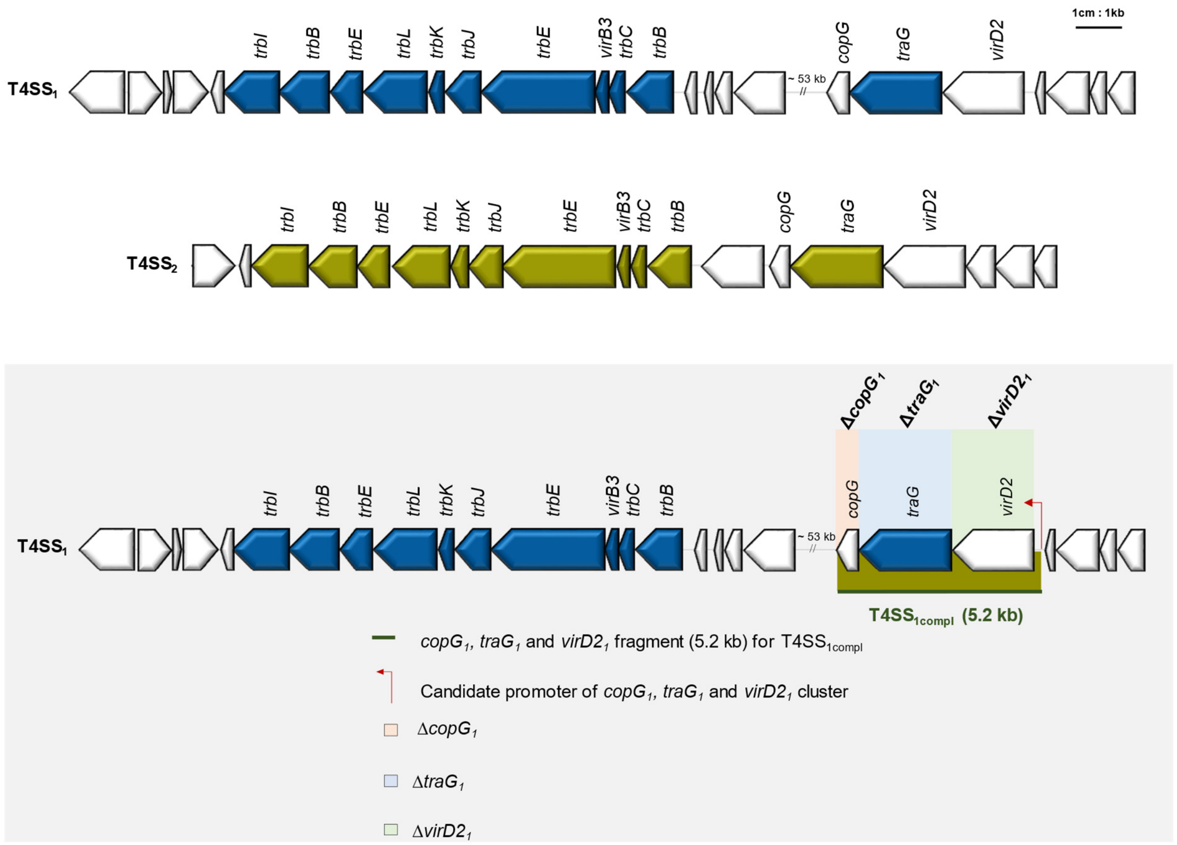

Figure S1. Genetic organization of type IV secretion system cluster 1 ($T4SS_1$) and cluster 2 ($T4SS_2$) on the chromosome of *Bradyrhizobium* sp. SUTN9-2. The characterizations of mutants are indicated by arrows and lines. Modified from Wangthaisong et al., 2023 [3].

```
CLUSTAL 2.1 multiple sequence alignment


copG1           CTAGTGGCCGTCCGCCTTCATTGTTTCTGGGCTGAATTGGACGCTTTCCACGTCATCCGG
copG2           -----------------TCAGGTTTCTTCAACCGGCTCCTG-ACTGCGGATATCTTCCGG
                                 ***   **  *   * *  *      **    *  ** *****

copG1           GATTTCCTGTCGCA-GCTTCGGCCCTTGACTCAGCCGGCGCCCGAGCGCCGCGACGAAAT
copG2           AATCTCGCGCAGAAAGCTTTGGCCCTTT-TGCAAGCGACGCCCAAGCGCCTCGACAAATC
                 ** **  *  * * **** *******    **  ** ***** ****** **** **

copG1           TGTCGTAGCGCGCACCTGCCTGCGCCCGCGCCGCCTTTGCAGCTGGTTCGGGGAGAGGCG
copG2           CCTCGAAGCGCTCCCTGCCCTTTGCCTGCGCGGCCGCCTGTGCATCGTTAGGCAACGGTG
                  *** ***** * *   ***  *** **** ***      **    *  ** *  ** *

copG1           GTGTCGTTGTGAGCCAGAAGCGGATGAAGAGAGCGATGGTCTCGACCGAAATTCCAACAT
copG2           GCGTAATAGTCAGCCAGAACCGAATGAACAGAGCCAAGGTCTCCGCCGTCAACCCAAGGT
                * **  * ** ******** ** ***** ***** * ******  ***  *  ****  *

copG1           CCCGCTCAAGCCGCGCGATCCGGCGGTCGATTTGGTCGAGTCGCTTGGCAATAGCGGCCT
copG2           CGCGCTCGAGCCTCTGCATCTGACGCGACAGCCGGTCGAGGCGCCGGGTAAACGCGGCCT
                * ***** **** *   *** * **    *   ******* ***  ** **  *******

copG1           CCCGCCGTTCATCGGAGTCGGGCGACAGAAAGGATGCAATGGCGGCTTCGGCAATAAGCG
copG2           CTCGCCGGTCAGCGCCGTCCGGCGATAGAAACGAAGCGACCGCAGCTTCCACGATCGCAG
                * ***** *** **  *** ***** ***** ** ** *  ** *****  * **    *

copG1           ACAGCGATTGCTCGCGCCGGGCGGCAAAGGCCGACAGCGCCTTCATGACATCAGGATCGA
copG2           ACCGAGAAAGCTTCTTGCGATCGGCAAGCTCCGAGATCTGTTTCAGAAGCTCTGGCGGGA
                ** * **  ***     **  ******   **** * *   ****  *  ** **   **

copG1           GATAGACCGACATCTGGGCTTTTCTCTTCGGAGCGGTCAT
copG2           AATAGACGTTCATTCGGTCGCGCAT---------------
                 ******   ***  ** *     *
```

CLUSTAL 2.1 Multiple Sequence Alignments

Sequence 1: *copG1*   459 bp

Sequence 2: *copG2*   426 bp

Sequences (1:2) Aligned. Score: 52.35

Figure S2. CLUSTALW (2.1) Multiple sequence alignments of *copG1* and *copG2* gene sequences in *Bradyrhizobium* sp. SUTN9-2. The definitions of the consensus colors are shown in black for

identical sequences and red for dissimilar sequences. The nucleotide sequences alignments were represented as follows: “*”, conserved sequences (identical), “–”, gap.

```
CLUSTAL 2.1 multiple sequence alignment


CopG1           MTAPKRKAQMSVYLDPDVMKALSAFAARREQSLSLIAEAAIASFLSPDSDERREAAIAKR
CopG2           -----MRDRMNVYFPPELLKQISELADRKKLSRSAIVEAAVASFLSPDGADRREAAFTRR
                      : :*.**: *:::* :* :* *:: * * *.***:*******. :*****:::*

CopG1           LDQIDRRIARLERDVGISVETIALFIRFWLTTTPPLPEPAAKAARAQAGARYDNFVAALG
CopG2           LDRLSRQMQRLERDLGLTAETLALFIRFWLTITPPLPNDAQAAAQAKGRERFEGFVEALG
                **::.*:: *****:*::.**:********* *****: *  **:*:.  *::.** ***

CopG1           RRLSQGPKLRQEIPDDVESVQFSPETMKADGH
CopG2           RRLQKGQSFLREIPEDIRSQEPVEET------
                ***.:* .: :***:*:.* :   **
```

CLUSTAL 2.1 Multiple Sequence Alignments

Sequence 1: $CopG_1$ 152 aa

Sequence 2: $CopG_2$ 141 aa

Sequences (1:2) Aligned. Score: 51.773

Figure S3. CLUSTALW (2.1) Multiple sequence alignments of $CopG_1$ and $CopG_2$ amino acid sequences in *Bradyrhizobium* sp. SUTN9-2. Definitions of consensus colors are shown in black for identical sequences and red for dissimilar sequences. The protein sequence alignments were represented as follows: “*”, conserved sequences (identical), “:”, conservative mutation, “·”, semi-conservative mutation, “–”, gap.

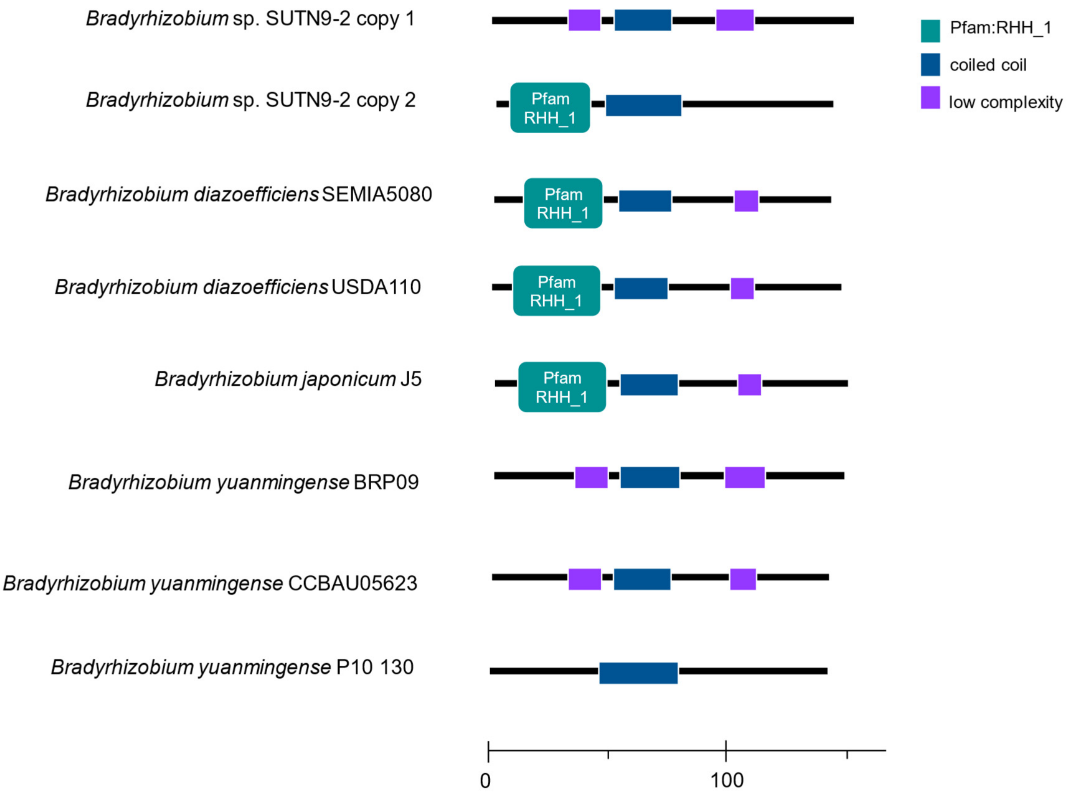

Figure S4. Protein domain architecture analysis of CopG proteins in bradyrhizobia. The colors of domain classifications are consistent with labeling.

MATRIX SCIENCE **MASCOT Search Results**

Protein View: A0A840HIL1|A0A840HIL1_9BRAD

Type III secretion translocon protein HrpF OS=Bradyrhizobium sp. ERR14 OX=2663837 GN=GGD62_006208 PE=4 SV=1

**Database:**	uniprotkb_Bradyrhizobium_sp_2023_06_29
**Score:**	1283
**Monoisotopic mass (M_r):**	64031
**Calculated pI:**	5.12

Sequence similarity is available as an NCBI BLAST search of A0A840HIL1|A0A840HIL1_9BRAD against nr.

Search parameters

**MS data file:**	D:\ID proteins\mgf\260\wt 1.mgf
**Enzyme:**	Trypsin/P: cuts C-term side of KR.
**Fixed modifications:**	Carbamidomethyl (C)
**Variable modifications:**	Oxidation (M)

Protein sequence coverage: 27%

Matched peptides shown in ***bold red***.

```
  1 MSVNNLSLTA SSSLSGLAPG PSSARDLSNF EAMLASFSLK DNVDDHSTPQ
 51 DPPAADSAKQ LTEELTQEVR NILPPEIKAA LDACQQAPEP SSAESSAVAS
101 APKIAEAPVQ SSRITWNSGT LTDAELQIVS VLNRHKDKCP LDWKSLGDLA
151 NDPSTPPDLK AAIEALQLDP ELFYAIGSQG DGRCGGKIKA GDLSGFSDHH
201 PQVAAFQEQR AKSYTQNYIP SDGTRNGEPS VMTQTDALRE LYRYSDNLPK
251 NLSLADFKRI VDGEAKTGKS PPQVLAAAQY FLDHPDEWKH LYGGSIDKVH
301 KEDFLQVASS SMNLSQAELD TLNTISKNQN AFFGKGDLTR EKLASMIDDK
351 SLPPNVRKAA SQLLSDPVLF GLMNNAITGY KTHHKFFDFG GGHTVDSGNI
401 SNTDFNQFFN NMSGANRTVH QVKTHAARTP AEQDAVADMT MGVADQPDIK
451 SPKKNGGAFM HALNDVLKVG SKMFDWAATA VGVLGFIPGL GELADLASMV
501 LEAEAQAANL LHTAISGGNM KQALEEAGLS LAAQAVGCIA GPEVKLAMRE
551 GLVKQAIQEA ATAGINLPVS VAQSYAEDYL NNLKARIESE RLQAAGANA
```

Unformatted sequence string: 599 residues (for pasting into other applications).

Sort by ◉ residue number ○ increasing mass ○ decreasing mass
Show ◉ matched peptides only ○ predicted peptides also
Show ◉ uncorrected delta ○ delta corrected for 13C

Query	Start – End	Observed	Mr(expt)	Mr(calc)	Delta	M	Score	Expect	Rank	U	Peptide
1113	71 – 78	462.2785	922.5424	922.5487	-0.0063	0	19	1.7	3		R.NILPPEIK.A
1603	104 – 113	529.2541	1056.4937	1056.5564	-0.0626	0	17	2	5		K.IAEAPVQSSR.I
1605	104 – 113	353.1912	1056.5518	1056.5564	-0.0046	0	58	0.0017	1		K.IAEAPVQSSR.I
1606	104 – 113	529.2872	1056.5599	1056.5564	0.0036	0	61	0.0025	1		K.IAEAPVQSSR.I
1623	137 – 144	354.5057	1060.4952	1060.5012	-0.0059	1	37	0.17	1		K.DKCPLDWK.S
2731	145 – 160	820.4061	1638.7976	1638.8101	-0.0124	0	157	1.2e-012	1		K.SLGDLANDPSTPPDLK.A
2732	145 – 160	410.7073	1638.8001	1638.8101	-0.0100	0	32	0.013	1		K.SLGDLANDPSTPPDLK.A
2733	145 – 160	547.2745	1638.8017	1638.8101	-0.0084	0	71	5e-005	1		K.SLGDLANDPSTPPDLK.A
2599	226 – 239	759.8569	1517.6993	1517.7144	-0.0151	0	95	2e-007	1		R.NGEPSVMTQTDALR.E
2600	226 – 239	506.9083	1517.7030	1517.7144	-0.0114	0	23	0.26	1		R.NGEPSVMTQTDALR.E
2602	226 – 239	760.3453	1518.6761	1517.7144	0.9617	0	43	0.0066	1		R.NGEPSVMTQTDALR.E
2603	226 – 239	760.3477	1518.6808	1517.7144	0.9664	0	80	1.5e-005	1		R.NGEPSVMTQTDALR.E
2605	226 – 239	507.2354	1518.6844	1517.7144	0.9700	0	56	0.00013	1		R.NGEPSVMTQTDALR.E
2624	226 – 239	767.8539	1533.6932	1533.7093	-0.0161	0	83	7e-007	1		R.NGEPSVMTQTDALR.E + Oxidation (M)
2625	226 – 239	512.2399	1533.6978	1533.7093	-0.0115	0	54	8e-005	1		R.NGEPSVMTQTDALR.E + Oxidation (M)
2626	226 – 239	512.5676	1534.6810	1533.7093	0.9717	0	45	0.0054	1		R.NGEPSVMTQTDALR.E + Oxidation (M)
2627	226 – 239	768.3495	1534.6844	1533.7093	0.9751	0	102	2.2e-007	1		R.NGEPSVMTQTDALR.E + Oxidation (M)
2628	226 – 239	512.5697	1534.6873	1533.7093	0.9780	0	62	0.00022	1		R.NGEPSVMTQTDALR.E + Oxidation (M)
740	244 – 250	418.7174	835.4203	835.4076	0.0128	0	45	0.0094	1		R.YSDNLPK.N
1047	251 – 258	454.2456	906.4767	906.4811	-0.0043	0	45	0.0067	1		K.NLSLADFK.R
1054	251 – 258	454.7339	907.4532	906.4811	0.9721	0	57	0.011	1		K.NLSLADFK.R
1628	251 – 259	532.2507	1062.4869	1062.5822	-0.0953	1	10	1.5	10		K.NLSLADFKR.I
1630	251 – 259	532.2926	1062.5706	1062.5822	-0.0115	1	37	0.46	1		K.NLSLADFKR.I
1631	251 – 259	355.2010	1062.5812	1062.5822	-0.0010	1	36	0.1	1		K.NLSLADFKR.I
1636	251 – 259	355.5260	1063.5561	1062.5822	0.9739	1	21	0.32	1		K.NLSLADFKR.I
1637	251 – 259	355.5276	1063.5611	1062.5822	0.9790	1	21	0.32	1		K.NLSLADFKR.I
1367	290 – 298	495.2569	988.4992	988.4978	0.0015	0	51	0.0078	1		K.HLYGGSIDK.V
1115	328 – 335	462.7793	923.5440	924.4454	-0.9013	0	48	0.0061	1		K.NQNAFFGK.G
1117	328 – 335	463.2358	924.4571	924.4454	0.0118	0	48	0.0058	1		K.NQNAFFGK.G
1118	328 – 335	463.2449	924.4752	924.4454	0.0299	0	18	1.3	2		K.NQNAFFGK.G
1122	328 – 335	463.7189	925.4233	924.4454	0.9779	0	31	0.048	1		K.NQNAFFGK.G
1123	328 – 335	463.7190	925.4235	924.4454	0.9781	0	16	0.29	1		K.NQNAFFGK.G
1124	328 – 335	463.7197	925.4249	924.4454	0.9796	0	23	0.19	1		K.NQNAFFGK.G
2943	359 – 381	813.7538	2438.2396	2438.2515	-0.0120	0	121	7.9e-011	1		K.AASQLLSDPVLFGLMNNAITGYK.T + Oxidation
2956	424 – 450	947.1028	2838.2866	2838.3276	-0.0411	1	126	1.3e-010	1	U	K.THAARTPAEQDAVADMTMGVADQPDIK.S
2957	424 – 450	710.5800	2838.2909	2838.3276	-0.0367	1	69	7e-006	1	U	K.THAARTPAEQDAVADMTMGVADQPDIK.S
2958	424 – 450	952.4340	2854.2801	2854.3226	-0.0425	1	136	2.7e-011	1	U	K.THAARTPAEQDAVADMTMGVADQPDIK.S + Oxida
2959	424 – 450	714.5775	2854.2807	2854.3226	-0.0419	1	83	1.2e-007	1	U	K.THAARTPAEQDAVADMTMGVADQPDIK.S + Oxida
2960	424 – 450	952.4368	2854.2885	2854.3226	-0.0341	1	110	3.4e-008	1	U	K.THAARTPAEQDAVADMTMGVADQPDIK.S + Oxida
2961	424 – 450	714.5818	2854.2981	2854.3226	-0.0245	1	87	2.8e-006	1	U	K.THAARTPAEQDAVADMTMGVADQPDIK.S + Oxida
2962	424 – 450	714.5839	2854.3065	2854.3226	-0.0161	1	36	0.003	1	U	K.THAARTPAEQDAVADMTMGVADQPDIK.S + Oxida
2963	424 – 450	957.7617	2870.2632	2870.3175	-0.0543	1	54	0.0015	1	U	K.THAARTPAEQDAVADMTMGVADQPDIK.S + 2 Oxi
2964	424 – 450	957.7698	2870.2876	2870.3175	-0.0299	1	133	1.9e-010	1	U	K.THAARTPAEQDAVADMTMGVADQPDIK.S + 2 Oxi
2965	424 – 450	718.5793	2870.2883	2870.3175	-0.0292	1	112	1.7e-009	1	U	K.THAARTPAEQDAVADMTMGVADQPDIK.S + 2 Oxi
2966	424 – 450	718.5801	2870.2913	2870.3175	-0.0262	1	55	0.00067	1	U	K.THAARTPAEQDAVADMTMGVADQPDIK.S + 2 Oxi
2935	522 – 545	794.7403	2381.1991	2381.2260	-0.0270	0	81	5.6e-007	1		K.QALEEAGLSLAAQAVGCIAGPEVK.L

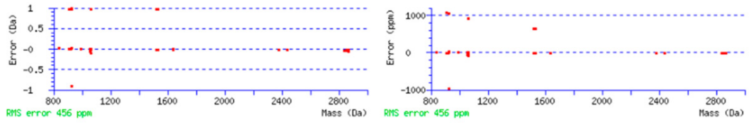

Figure S5. Amino acid sequences of protein selected band 1, analyzed by the MASCOT program.

MATRIX SCIENCE MASCOT Search Results

Protein View: A0A4Q0QMT4|A0A4Q0QMT4_9BRAD

C4-dicarboxylate ABC transporter OS=Bradyrhizobium vignae OX=1549949 GN=EAV90_23715 PE=3 SV=1

**Database:**	uniprotkb_Bradyrhizobium_sp_2023_06_29
**Score:**	306
**Monoisotopic mass (M_r):**	36417
**Calculated pI:**	8.86

Sequence similarity is available as an NCBI BLAST search of A0A4Q0QMT4|A0A4Q0QMT4_9BRAD against nr.

Search parameters

**MS data file:**	D:\ID proteins\mgf\260\wt 2.mgf
**Enzyme:**	Trypsin/P: cuts C-term side of KR.
**Fixed modifications:**	Carbamidomethyl (C)
**Variable modifications:**	Oxidation (M)

Protein sequence coverage: 31%

Matched peptides shown in ***bold red***.

1 MITRRHLIAT AVAAPAILRF GTGTAQAATT LK**ISHQFPGG TIDK**GDFRDR
51 LCRMFAAEVA KR**SNGDIAAE IYPNSSLIKT NAQFSAMR**KG ALDISLYPMP
101 YAGGELPETN IGLMPGLVTT YDQGLRWKKE PVGK**ALTDFL ADK**GIILLTW
151 VWQAGGVASR **SKPIVAPEDA KGMKVRGGSR** EMDMVLQTAG ASVLSVPSNE
201 IYAAMQTGAC DAGITSSTSL ISFRLEEVAK SLTSGAGTSY WFMLEPLMMS
251 K**AIFDKLPK**N HQDILLAVGT ELEAFGR**KGA QDDDVEVAK**V YEKAGAK**VSA**
301 **LDAATVGK**WR DIARDTAWKD YSAK**TATAAN LLK**LAVDVAA

Unformatted sequence string: 340 residues (for pasting into other applications).

Sort by ◉ residue number ○ increasing mass ○ decreasing mass
Show ◉ matched peptides only ○ predicted peptides also
Show ◉ uncorrected delta ○ delta corrected for 13C

Query	Start – End	Observed	Mr(expt)	Mr(calc)	Delta	M	Score	Expect	Rank	U	Peptide
1577	33 – 44	433.8906	1298.6500	1298.6619	-0.0119	0	40	0.0016	1	U	K.ISHQFPGGTIDK.G
1942	63 – 79	597.9701	1790.8885	1790.9050	-0.0165	0	65	6.6e-005	1	U	R.SNGDIAAEIYPNSSLIK.T
1010	80 – 88	513.2402	1024.4659	1024.4760	-0.0101	0	46	0.0083	1	U	K.TNAQFSAMR.K
1048	80 – 88	521.2374	1040.4602	1040.4709	-0.0107	0	11	1.3	4	U	K.TNAQFSAMR.K + Oxidation (M)
924	135 – 143	497.2612	992.5079	992.5179	-0.0099	0	65	0.0016	1	U	K.ALTDFLADK.G
1329	161 – 171	385.5505	1153.6296	1153.6343	-0.0046	1	48	0.0025	1	U	R.SKPIVAPEDAK.G
1528	163 – 174	635.8357	1269.6568	1270.6591	-1.0023	1	9	1.7	8	U	K.PIVAPEDAKGMK.V + Oxidation (M)
801	172 – 180	475.2754	948.5362	946.5131	2.0232	2	5	1.1	2	U	K.GMKVRGGSR.E
749	252 – 259	466.2796	930.5446	930.5538	-0.0092	1	34	0.11	1	U	K.AIFDKLPK.N
1538	278 – 289	637.8084	1273.6021	1273.6150	-0.0129	1	82	1.4e-005	1	U	R.KGAQDDDVEVAK.V
1539	278 – 289	425.5432	1273.6076	1273.6150	-0.0074	1	83	6.5e-006	1	U	R.KGAQDDDVEVAK.V
1311	279 – 289	573.7616	1145.5086	1145.5201	-0.0114	0	105	1.8e-007	1	U	K.GAQDDDVEVAK.V
1030	298 – 308	516.2880	1030.5614	1030.5659	-0.0045	0	83	7e-006	1	U	K.VSALDAATVGK.W
650	325 – 333	451.7651	901.5157	901.5233	-0.0075	0	64	0.0028	1	U	K.TATAANLLK.L

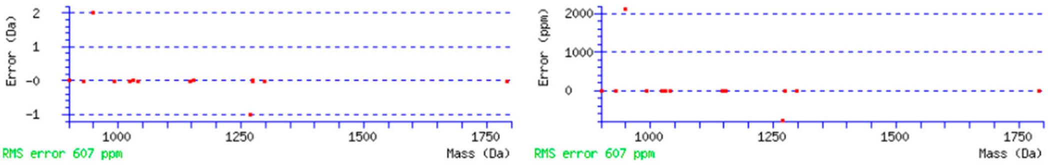

Figure S6. Amino acid sequences of protein selected band 3, analyzed by the MASCOT program.

MATRIX SCIENCE MASCOT Search Results

Protein View: A0A3N6MRZ5|A0A3N6MRZ5_9BRAD

Host specificity protein OS=Bradyrhizobium sp. RP6 OX=2489596 GN=EHH60_33850 PE=4 SV=1

**Database:**	uniprotkb_Bradyrhizobium_sp_2023_06_29
**Score:**	120
**Monoisotopic mass (M_r):**	25509
**Calculated pI:**	4.88

Sequence similarity is available as an NCBI BLAST search of A0A3N6MRZ5|A0A3N6MRZ5_9BRAD against nr.

Search parameters

**MS data file:**	D:\ID proteins\mgf\260\wt 3.mgf
**Enzyme:**	Trypsin/P: cuts C-term side of KR.
**Fixed modifications:**	Carbamidomethyl (C)
**Variable modifications:**	Oxidation (M)

Protein sequence coverage: 18%

Matched peptides shown in ***bold red***.

```
  1 MDSDEFGEEV KAFYGDDIKY IAERPLEYSD FISTKAERAA NIARTYGHTD
 51 EDTDQARYYA YRLGDKTVGL LRTEGGLRVG GRRFSAQFPG RNAVTSQVDL
101 RVTHPLVENA GDILLEHQLR QDGEQALIMS KPAFPGIESR LAQMGFVPVS
151 DKNHWVLDPH QHPDKWTHND EGKWQRVGKP EGYLAAESSG AQANIERASS
201 EASDETESSG DDTSWYFERL NLGPGSAAE
```

Unformatted sequence string: 229 residues (for pasting into other applications).

Sort by ◉ residue number ○ increasing mass ○ decreasing mass
Show ◉ matched peptides only ○ predicted peptides also
Show ◉ uncorrected delta ○ delta corrected for 13C

Query	Start - End	Observed	Mr(expt)	Mr(calc)	Delta	M	Score	Expect	Rank	U	Peptide
839	45 - 57	503.5434	1507.6084	1507.6175	-0.0092	0	78	1.1e-005	1	U	R.TYGHTDEDTDQAR.Y
373	84 - 91	455.2303	908.4460	908.4505	-0.0044	0	41	0.029	1	U	R.FSAQFPGR.N
758	121 - 131	618.2914	1234.5682	1234.5863	-0.0181	0	83	5.9e-006	1	U	R.QDGEQALIMSK.P + Oxidation (M)
410	220 - 229	464.7365	927.4585	927.4661	-0.0076	0	27	0.95	1	U	R.LNLGPGSAAE.-

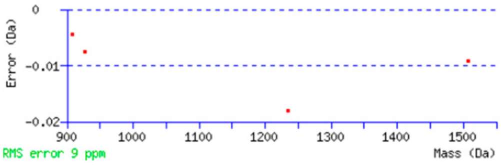

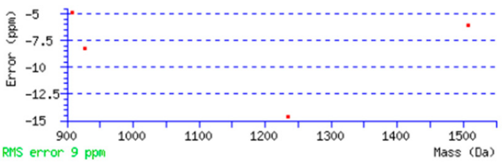

Figure S7. Amino acid sequences of protein selected band 6, analyzed by the MASCOT program.

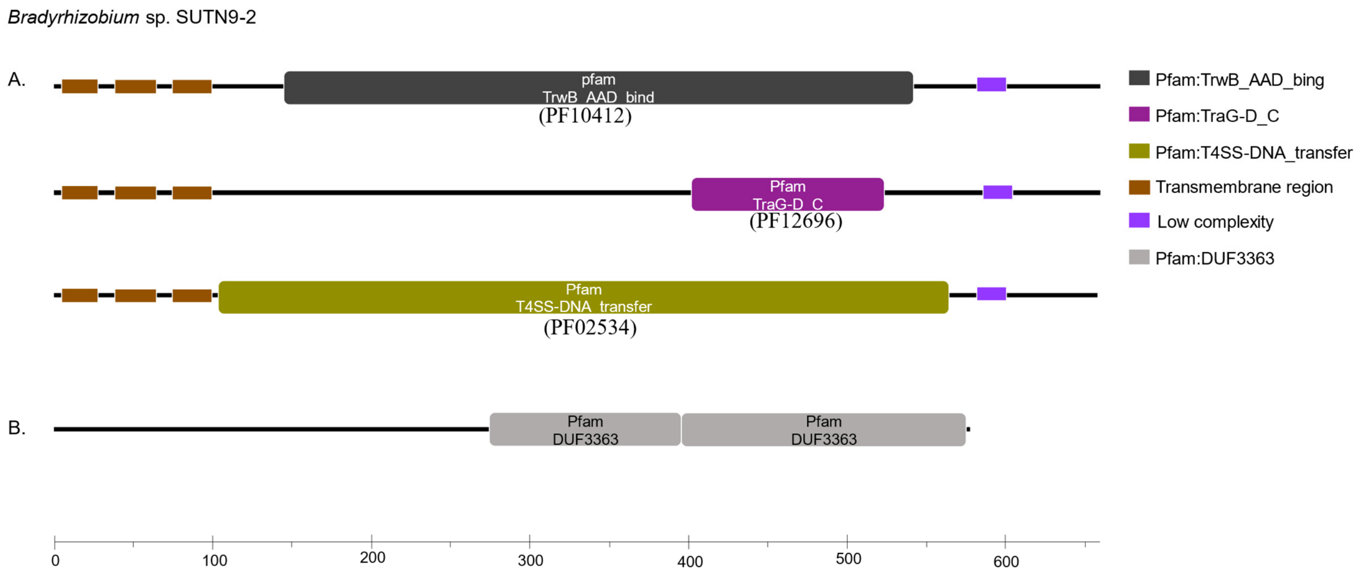

Figure S8. Protein domain architecture analysis of TraG$_1$ (A) and VirD2$_1$ (B) protein sequences in *Bradyrhizobium* sp. SUTN9-2. The colors of domain classifications are consistent with labeling.

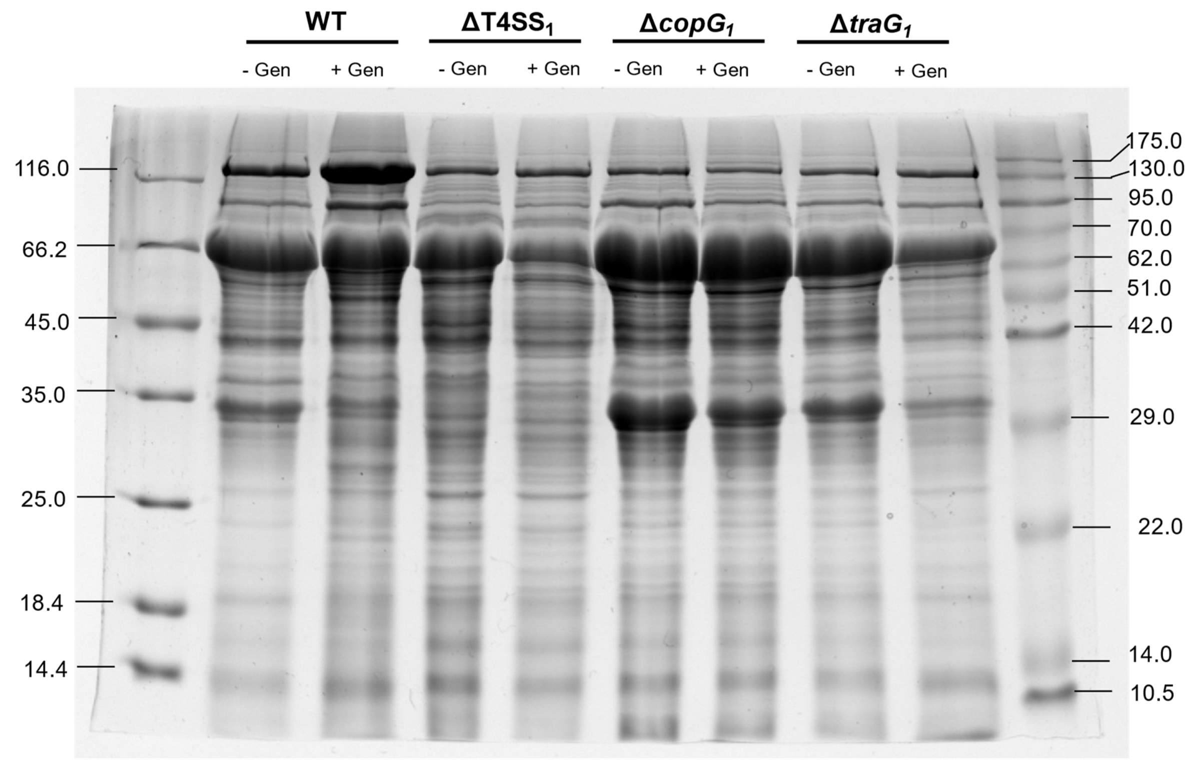

Figure S9. Original SDS-PAGE of proteins secretion into the external medium of *Bradyrhizobium* sp. SUTN9-2 (WT) and mutant strains with 20 µM genistein (+Gen) and without 20 µM genistein (Gen) induction. Numbers on the left indicate molecular size markers in kilodalton.

Reference

1. Ditta, G.; Stanfield, S.; Corbin, D.; Helinski, D.R. Broad host range DNA cloning system for Gram-negative bacteria: construction of a gene bank of *Rhizobium meliloti*. *Proc. Natl. Acad. Sci.* **1980**, *77*, 7347–7351.
2. Tsai, J.-W.; Alley, M.R.K. Proteolysis of the McpA chemoreceptor does not require the Caulobacter major chemotaxis operon. *J. Bacteriol.* **2000**, *182*, 504–507.
3. Wangthaisong, P.; Piromyou, P.; Songwattana, P.; Wongdee, J.; Teamtaisong, K.; Tittabutr, P.; Boonkerd, N.; Teaumroong, N. The type IV secretion system (T4SS) mediates symbiosis between *Bradyrhizobium* sp. SUTN9-2 and legumes. *Appl. Environ. Microbiol.* **2023**, *89*, e00040-23.
